# Bat point counts: A novel sampling method shines light on flying bat communities

**DOI:** 10.1002/ece3.8356

**Published:** 2021-11-30

**Authors:** Kevin Felix Arno Darras, Ellena Yusti, Joe Chun‐Chia Huang, Delphine‐Clara Zemp, Agus Priyono Kartono, Thomas Cherico Wanger

**Affiliations:** ^1^ Agroecology Department of Crop Sciences University of Göttingen Göttingen Germany; ^2^ Sustainable Agriculture & Technology Lab School of Engineering Westlake University Hangzhou China; ^3^ EFForTS University of Jambi Jambi Indonesia; ^4^ Southeast Asian Bat Conservation Research Unit Lubbock Texas USA; ^5^ Biodiversity, Macroecology and Biogeography University of Göttingen Göttingen Germany; ^6^ Laboratory of Conservation Biology Institute of Biology University of Neuchâtel Neuchâtel Switzerland; ^7^ Department of Forest Resources Conservation and Ecotourism Faculty of Forestry IPB University Bogor Indonesia; ^8^ Key Laboratory of Coastal Environment and Resources of Zhejiang Province Westlake University Hangzhou China; ^9^ GlobalAgroforestryNetwork.org China

**Keywords:** biodiversity sampling, Chiroptera, near‐infrared, point count, thermal, ultrasound

## Abstract

Emerging technologies based on the detection of electro‐magnetic energy offer promising opportunities for sampling biodiversity. We exploit their potential by showing here how they can be used in bat point counts—a novel method to sample flying bats—to overcome shortcomings of traditional sampling methods, and to maximize sampling coverage and taxonomic resolution of this elusive taxon with minimal sampling bias. We conducted bat point counts with a sampling rig combining a thermal scope to detect bats, an ultrasound recorder to obtain echolocation calls, and a near‐infrared camera to capture bat morphology. We identified bats with a dedicated identification key combining acoustic and morphological features, and compared bat point counts with the standard bat sampling methods of mist‐netting and automated ultrasound recording in three oil palm plantation sites in Indonesia, over nine survey nights. Based on rarefaction and extrapolation sampling curves, bat point counts were similarly effective but more time‐efficient than the established methods for sampling the oil palm species pool in our study. Point counts sampled species that tend to avoid nets and those that are not echolocating, and thus cannot be detected acoustically. We identified some bat sonotypes with near‐infrared imagery, and bat point counts revealed strong sampling biases in previous studies using capture‐based methods, suggesting similar biases in other regions might exist. Our method should be tested in a wider range of habitats and regions to assess its performance. However, while capture‐based methods allow to identify bats with absolute and internal morphometry, and unattended ultrasound recorders can effectively sample echolocating bats, bat point counts are a promising, non‐invasive, and potentially competitive new tool for sampling all flying bats without bias and observing their behavior in the wild.

## Graphical abstract

Bat point counts are a modern, novel sampling method using thermal, ultrasonic, and near‐infrared sensors. It rivals established mist‐netting and passive acoustic monitoring methods. We showcase the unique advantages of the method offers and how it shines a new light on all types of flying bats.

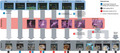

## 1 INTRODUCTION

Biodiversity sampling is biased toward species that are easily and directly detectable with our human senses (Moussy et al., 2021). Even though remote visible light imagery has been used for decades (Blackwell et al., 2006; Cutler & Swann, 1999), newer technologies based on the detection of the broader electromagnetic energy spectrum are becoming more accessible and further facilitate detecting and identifying animals passively and remotely (Turner et al., 2003; Vance et al., 2016). The applications in ecology and biodiversity conservation have great potential for scientists and conservationists (Pimm et al., 2015), especially when sampling elusive animals. Here, we focus on the detection of bats (Chiroptera), a taxon that is notoriously difficult to sample because they are nocturnal, fast, and silent fliers. This partly explains the relative lack of knowledge about bats, although they are the second most diverse order of mammals, they provide important, wide‐ranging ecosystem services, and they experience acute threats (Frick et al., 2020; Kunz et al., 2011).

Bats are typically studied by capture using traps or by roost surveys. Mist‐netting and harp‐trapping are the most common sampling methods for bats outside of their roosts. They are used to describe bat communities and are also valuable for measuring the bats’ morphology precisely, taking physical samples (blood, tissue, parasites), assessing their physiological status, and estimating bat abundance directly. However, they are logistically challenging and have biases: species flying above nets (e.g., large fruit bats) are rarely caught, nets are avoided by some echolocating bats (e.g., “whispering bats”), and other bats can learn to avoid them, requiring daily net moving (Marques et al., 2013). Harp traps are more effective for some species, but they have variable performance (Berry et al., 2004) and may be more useful in South‐east Asia (Furey et al., 2010). Furthermore, permits are often needed for catching bats, their handling comes with potential zoonotic risks (Wong et al., 2007), and the animals become stressed and more vulnerable to predation (Rocha‐Mendes & Bianconi, 2009); they can even succumb to this invasive sampling method.

Passive acoustic monitoring is also commonly used for sampling bats, since most bats vocalize in the ultrasonic range for navigation with so‐called echolocation calls. Passive ultrasound recording relies on automated devices to record echolocation calls. Single, affordable devices can sample large spaces and be programmed to record for long durations. However, the vast majority of Pteropodidae, occurring in the Paleotropics and Oceania, do not echolocate (except genus *Rousettus*), which explains why capture‐based methods are essential there. Still, little is known about bat acoustics in the tropics, and acoustic methods need to be adopted more widely, especially in the Paleotropics (Kingston, 2010). Also, bats do not necessarily have species‐specific echolocation calls, and calls are variable (Obrist, 1995). As a result, many species cannot be distinguished on the basis of ultrasound alone and are grouped within “sonotypes” (Walters et al., 2013). Finally, very high frequency bat calls usually attenuate quickly in air and are seldom picked up by microphones that have declining sensitivity with frequency. Some bats also produce narrow ultrasound beams which are less likely to hit a microphone (Brinkløv et al., 2011). Finally, sound detection spaces are species‐specific and seldom accounted for (Darras et al., 2016). Thus, acoustic detection and identification of bats is challenging, and density estimation is nearly impossible—especially across species.

Mist‐netting and passive acoustic monitoring are now established, standardized sampling methods for bat biodiversity surveys (Flaquer et al., 2007). It is often advised to combine both methods to reduce the overall sampling bias (Kuenzi & Morrison, 1998), especially where Pteropodidae occur. However, recently, a proof‐of‐concept has been proposed for technologically enabled point counts to sample flying bats at night (Darras et al., 2021). These bat point counts are an active (i.e., requiring a human operator) sampling method to detect and identify all flying bats within a sampling area at night, combining thermal sensing to detect flying bats, ultrasound sampling to record their echolocation calls, and near‐infrared imagery to capture their morphology. Thermal and near‐infrared imagery have been used before to count bat colonies directly in caves (Betke et al., 2008; Sabol & Hudson, 1995), and thermal imaging has also been combined with ultrasound recording to detect bats with drones (Fu et al., 2018) and at wind farms (Correia et al., 2013). Near‐infrared imaging can also detect pollinating bats (Frick et al., 2009). However, these studies surveyed precise locations with a great density of bats or a high probability of encountering them. Near‐infrared imaging has not been used yet for identifying flying bats over large areas. This becomes possible when combined with thermal imaging—as to enable efficient, passive detection of homeotherms—and ultrasound recording—as to support the discrimination of morphologically similar species. However, it remains to be seen whether entire bat communities can be sampled with this method and how it compares to established methods.

Here, we showcase bat point counts and demonstrate how they can be used for ecological studies. We compare them with mist‐netting and ultrasound recording in an agricultural system in the Paleotropics, where both insectivorous, echolocating bats and frugivorous, non‐echolocating bats are common. We measure the detection spaces of all three sampling methods, present a novel, morphological‐acoustic bat identification key tailored to our study system to make use of the acoustic and photographic data, investigate how accurately and efficiently the species pools are sampled by each method, and compare diversity patterns using rarefaction and extrapolation sampling curves. We discuss practical considerations, and we give an outlook as to the new possibilities offered by bat point counts for the study of bats.

## 2 MATERIALS AND METHODS

### 2.1 Study site and design

**FIGURE 1:**
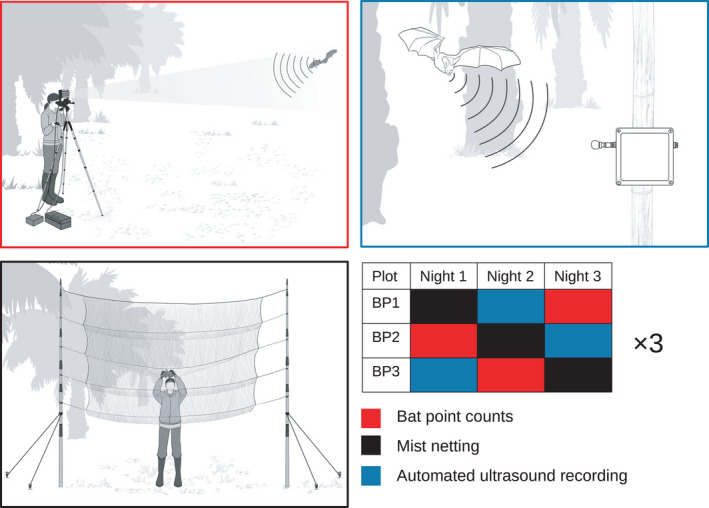
Illustrations of bat sampling methods and sampling schedule. Bat point counts were compared simultaneously against automated ultrasound recording and mist‐netting in three oil palm plantation sites (Plots BP1, BP2, and BP3) for nine nights. Drawings by JABU studio

We surveyed bats in three different sites in a closed‐canopy oil palm plantation using bat point counts, mist nets, and automated ultrasound recorders. Our sampling sites are inside the Humusindo Makmur Sejati (01.95°S and 103.25°E, 47 ± 11 m.a.s.l.) company estate, near Bungku village in the lowlands of Jambi province, Sumatra, Indonesia. We set the center of each site within 10 m of a stream (2–4 m wide) and an unpaved road (4–5 m wide) in order to maximize potential species detections, as it is widely known that bats use trails and streams for commuting and hunting (Voigt & Kingston, 2016). The sites were bordering the same river and separated by at least 600 m to allow independent captures. We sampled all three sites simultaneously with rotating methods on three consecutive nights with one field team, and we repeated this twice, obtaining three sampling nights for each method and site in total (Figure 1). The surveys occurred during nine nights from 21 to 31 May 2019. Due to our selection of sites with identical surrounding habitats, the temporally rotating design of the methods, the simultaneous comparison of methods in equivalent sites, and the short sampling period, any effects of weather conditions, moon phase, and fluctuating food resource availability was minimized and should not bias our results.

### 2.2 Bat point counts

We conducted bat point counts for 1 h per night in each site. We used a sampling rig with sensors for ultrasound, thermal, and near‐infrared waves; we provide full technical details of the rig and the observation workflow in (Darras et al., 2021). Each 10‐min point count scanned a 120° field of view, directed either toward the road, river, or oil palm. We determined the detection area of the full‐spectrum microphone [Parus open‐source model, (Darras et al., 2018)] fitted with a horn to amplify sounds from the front—with a chirp emitter at 40 kHz and the detection area of the thermal scope (Darras et al., 2021). The first three point counts took place in the first hour after the survey started, and the next three point counts happened in the second hour. Between the two hours, the bat point count team assisted bat extraction at the mist‐netting site that was permanently attended by a third person. Thus, it was not possible to sample bats with point counts for the same duration as with the other methods.

### 2.3 Mist‐netting

We mist‐netted bats for 4 h per night for a total of 576 net‐hours (m × h) in each site. Mist‐ netting was our reference trapping method for sampling bats: we did not use harp traps as they were ineffective in previous assessments in oil palm plantations in our region (Kevin Felix Arno Darras, K.F.A.D., unpublished data). We opened four 3.2 m high × 12 m long nets 1 to 1.5 m above the ground for four hours starting at sunset (Ultra Thin Series M Mist Net, 20 mm mesh, Ecotone). Nets were installed in presumed flight ways, delimited a quadrilateral, and their position relative to the river, oil palm, or road was approximately the same in all plots for each survey set. Most of the below‐canopy flying space was covered with our nets. Mist nets were checked every 15 min from sunset to 2 h afterward, then every 30 min until 4 h after sunset. Captured bats were kept in tissue pouches until the nets were closed. Bat morphology was measured to identify bats according to Huang et al. (2014) directly in the field. There were no particular regulations or ethical guidelines for research on live bats in Indonesia at the time of the study, but we wore protective equipment (masks and gloves) to handle them.

### 2.4 Ultrasound recordings

We made continuous, full‐spectrum ultrasoundscape recordings (i.e., without triggers) with sound recorders for 4 h per night in each site. One recorder (SM2Bat+, Wildlife Acoustics) was set up with one microphone [Parus open‐source model, (Darras et al., 2018)] parallel to the ground, sampling at 384 kHz at 2 m height, starting at sunset, and lasting 4 h in each site. We measured the ultrasound detection space covered by the recorders (Darras et al., 2016): similarly to bat point counts, we pointed an ultrasound calibrator (Wildlife Acoustics) to the recorder, and recorded its 40 kHz chirps emitted from 2 m height at distances of 4, 8, 16, and 32 m, in three directions (to the river, the road, and the oil palm plantation (because the microphone without horn is approximately omni‐directional) to derive the site's sound transmission profiles (Figure S1).

### 2.5 Data analysis

#### 2.5.1 Species identification

Ultrasound recordings from bat point counts and automated ultrasound recorders were uploaded on the open‐source platform BioSounds: https://biosounds.uni‐goettingen.de/ (Darras et al., 2020) to annotate the spectrograms with identified bat detections; we included both acoustic as well as thermal‐only detections (detections without ultrasound that were vocally mentioned by the observer) (Darras et al., 2021). All bat calls were identified using our reference collection of bat calls obtained from captured bats [Chiroptera reference collection in BioSounds (Darras et al., 2020)] and literature data (Hughes et al., 2011; Kingston, 2013; Zhu et al., 2012). We distinguished broadband frequency‐modulated (BFM), constant‐frequency (CF), and frequency‐modulated, quasi‐constant frequency (FM‐QCF) calls. We measured calls for each bat call type within each recording, but only if the bat pass was recorded clearly (to avoid biased call parameters from distant calls), using the three strongest, not saturated calls: We measured peak frequency (FmaxE, frequency with maximum energy), start frequency, end frequency, call duration, and inter‐pulse interval (from the start of one call to the onset of the next). Frequency‐modulated quasi‐constant frequency (FM‐QCF) calls were split in three sonotypes based on their end frequency: around 33 kHz, between 38 and 42 kHz, and around 48 kHz, because information in published data is insufficient to identify calls to species for Sumatra.

We matched ultrasound recordings and near‐infrared photographs from bat point counts to their respective detections using a conservative workflow and discarded thermal detections without photos or ultrasound (Darras et al., 2021). We used data from one additional, incomplete survey night (during which our infrared lamp power supply failed) to aid with the taxonomic identification. Bat species identification usually relies on direct, external, and internal body part measurements and categorical features of caught specimens. However, absolute measurements from pictures are inaccurate due to our large depth of field of approximately four meters: a 10 cm bat at a distance of 8 m would appear as large as a 15 cm bat at 12 m. Thus, we rely on categorical features as well as relative measurements of external, readily recognizable body parts—as they do not depend on the bats’ distance—for identifying bat point count detections. We used pixel‐measuring software tools on photos where the measured body parts were parallel to the camera focus plane to avoid underestimates. We only used near‐infrared images to identify thermal‐only detections. We confirmed or determined the identity of bat sonotypes in bulk by using clear near‐infrared pictures of selected detections. Based on this identification process, we devised a new identification key for South‐east Asian bats found in oil palm derived from Huang et al. (2014) to determine bat identity from near‐infrared pictures and ultrasound calls (Box 1).

BOX 1 **Identification key for Sumatran bats occurring in oil palm plantations, based on categorical and relative morphological and acoustic features. The key is adapted to the current highest quality of near‐infrared imagery obtained from our bat point counts. The species list is based on our own checklist of bats sampled using mist nets in oil palm plantations (Darras, unpublished data). FmaxE, frequency of maximal (i.e., peak) frequency**

**Table:** 

We discriminate Pteropodidae from other bat families based on their morphological adaptation and behavior. 1.1 Pteropodidae have visibly enlarged eyes with a retroreflective layer (tapetum lucidum) to see in very low light levels (Müller et al., 2007; Ollivier et al., 2004). Their intrafemoral membrane and tail are inconspicuous, and they have a straighter flight compared withinsectivorous bats that maneuver to hunt (EY, pers. obs.). → 2 1.2 Echolocating bats have characteristically small eyes, as they rely primarily on their auditory sense to navigate and forage. →5 We check the relative snout size using the head length (from back to snout tip) to head width (from throat to top) ratio: 2.1 Relatively short (ratio < 1.7) and robust snout →3 2.2 Relatively long (ratio ≥ 2) and narrow snout→4 We check for diagnostic features of different pteropodid genera: 3.1 Whitish digits (adult *Cynopterus*) 3.2 Spotted wings (*Balionycteris maculata*) 3.3 None of the above (*Megaerop*s/juvenile *Cynopterus*) We check for the overall body size by comparison with photographed habitat features: 4.1 Very large (*Pteropus*) 4.2 Intermediate or small (*Eonycteris*/*Rousettus*/*Macroglossus*) We distinguish echolocating families based on the relative size of the ears. Large ears are characteristic for bats passively listening for prey and are used to amplify the received ultrasound echoes (Obrist et al., 1993). 5.1 Ears approximately as large as the head, FM calls (Nycteridae, Megadermatidae) →6 5.2 Ears about half as large as the head or smaller →7 We use the tail to discriminate between both families: 6.1 Interfemoral membrane obvious, tail inconspicuous, FmaxE 58 kHz (*Megaderma spasma*) 6.2 Both interfemoral membrane and tail obvious, FmaxE 97 kHz (*Nycteris tragata*) We distinguish several families from their tail and interfemoral membrane shape: 7.1 Interfemoral membrane small, tail shorter than hind feet, ears half as large as head, nostrils open roughly perpendicularly to the open mouth, CF calls (*Rhinolophus*, *Hipposideros*) →8 7.2 Obvious tail extending from the interfemoral membrane (Molossidae, Emballonuridae, Rhinopomatidae) 7.3 Tail enclosed in obvious interfemoral membrane, ears less than one third of the head, snout direction points in similar direction as the mouth (Vespertilionidae, Miniopteridae) →9 Several species can be distinguished from their calls’ frequency of maximum energy: 8.1 FmaxE 78 kHz (*Hipposideros orbiculus*) 8.2 Fmax 137 kHz (*Hipposideros kunzi*) 8.3 Fmax 65 kHz (*Rhinolophus sedulus*) 8.4 Fmax 54 kHz (*Rhinolophus trifoliatus*) Vespertilionidae and Miniopteridae can be distinguished from the relative sizes of the phalanges of the third digit. 9.1 First phalange <40% of second phalange (Miniopteridae) 9.2 First phalange about as long as second phalange (Vespertilionidae)

#### 2.5.2 Rarefaction and extrapolation sampling curves

We compared the species richness sampling effectiveness and efficiency of bat point counts against mist‐netting and automated ultrasonic recording by comparing rarefaction and extrapolation sampling curves (Chao et al., 2014). We pooled all the three sampling sites to represent the bat community in our oil palm plantation. We calculated a conservative estimate of each species’ abundance in sound recordings by using the maximum number of simultaneously recorded bats per night. We computed taxon presence and absence at each sampling hour and method as well as abundance matrices for each method, by summing the species abundances over the three sites. We only used the taxonomic identities yielded by each sampling method, independently of the insights gained from the other methods. We generated raw incidence as well as abundance‐based rarefaction and extrapolation sampling curves using the iNEXT package in R (Hsieh et al., 2016) and compared the species richness (Hill number q = 0) at a 95% sampling coverage for a robust estimation of diversity (Chao & Jost, 2012). The R script and markdown report (Data S1) are available in the supplementary materials. Raw incidence‐based curves enabled to assess the temporal efficiency of each method by plotting species richness against the number of sampled hours; abundance‐based curves enabled to assess the workload efficiency by plotting species richness against the number of sampled individuals. We also generated coverage‐based rarefaction and extrapolation sampling curves (Data S2) but did not include them in the main text as sampling coverage is not indicative of sampling effectiveness. We assessed differences in species richness between sampling methods with 83% confidence intervals to detect significant (*p* < .05) differences between the estimated means graphically (Krzywinski & Altman, 2013).

#### 2.5.3 Acoustic and thermal detection ranges

Thermal detection ranges of bat point counts were obtained for each direction by measuring the maximal distance at which the hand of a field assistant (always the same) was detectable in the thermal scope. Acoustic detection ranges for point counts (with ultrasonic horn) and automated ultrasound recordings (without horn) were obtained for each site and direction from the intersection of the chirp emitter's sound pressure level profile—fit with a linear model against log‐transformed distance—with the ambient sound level (Darras et al., 2016). In contrast, sampling spaces of mist nets are not determinable *per se*, but we assumed that they cover at least the inner area delimited by their border.

## 3 RESULTS

### 3.1 Detected species

**FIGURE 2:**
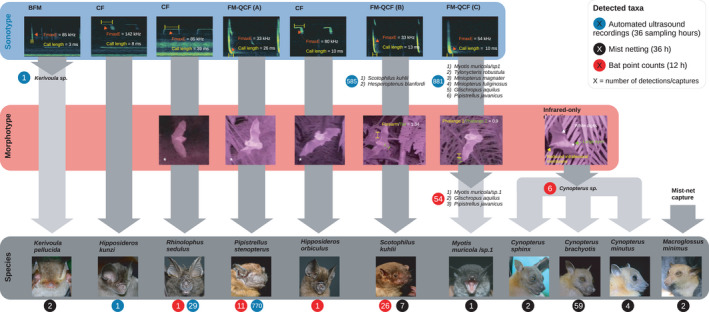
Identification workflow of bat taxa sampled in oil palm plantations in Sumatra (Indonesia) with bat point counts, as well as traditional mist‐netting and ultrasound recording methods. Call interval, start and end frequency were also used for the identification but are not shown here. Representative near‐infrared photographs are shown; they belong to sequences of multiple pictures. Asterisks denote near‐infrared imagery that was not strictly needed for identification but that was used for identity confirmation. Putative identification pathways are shown with a lighter gray tone. Photos used with permission from Ellena Yusti, Joe Chun‐Chia Huang, and Neil Jun Lobite

We found 100 thermal detections of six taxa in point counts, 2009 detections of six taxa in automated ultrasonic recordings, and captured 83 bats from seven species in mist nets (Figure 2). We excluded eight point count detections where neither ultrasound nor near‐infrared pictures were recorded. We found one BFM, three CF, and three FM‐QCF sonotypes (Table S1, Figure S2). We identified the BFM sonotype to genus in automated ultrasound recordings and found its putative identity from mist‐netting data (*Kerivoula pellucida*). We identified all CF sonotypes to species (*Hipposideros kunzi*, *Hipposideros orbiculus*, *Rhinolophus sedulus*) and none was found using mist nets. All FM‐QCF sonotypes were found using bat point counts and automated ultrasound recordings. One FM‐QCF was identified to species using acoustic data [*Pipistrellus stenopterus*, (Kingston, 2013)] and was not found in mist nets. Using relative measurements from near‐infrared imagery, one FM‐QCF sonotype consisting of two candidate species was resolved to species‐level in point counts by measuring the ratio between the forearm and tail lengths (*Scotophilus kuhlii*); it was the only species detected by all methods. The third FM‐QCF sonotype was a complex of six candidate species and was reduced to three candidate species using near‐infrared imagery. It was putatively identified with mist‐netting data [undescribed *Myotis* sp.1 *sensu* (Huang et al., 2014). One pteropodid genus (*Cynopterus*) was detected in bat point counts and resolved to three distinct species in the mist‐netting dataset (*Cynopterus sphinx*, *C. brachyotis*, *C. minutus*). Mist nets detected one pteropodid species from another genus (*Macroglossus minimus*).

### 3.2 Rarefaction and extrapolation sampling curves

**FIGURE 3:**
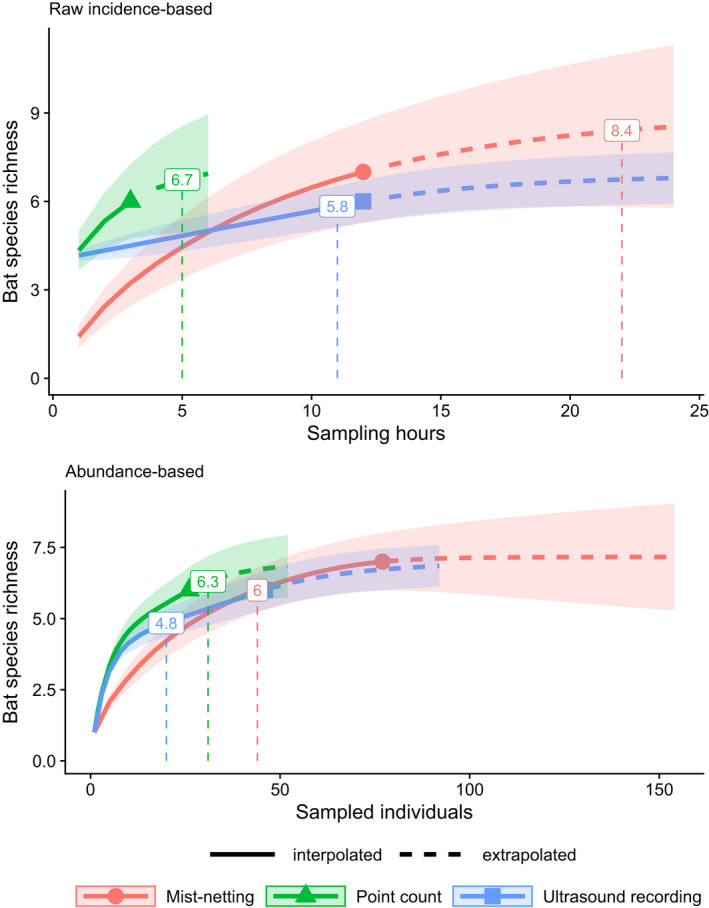
Rarefaction and extrapolation sampling curves for bat point counts, compared with established bat sampling methods. Shaded areas show 83% confidence intervals; differences in species richness are statistically significant when they do not overlap (Krzywinski & Altman, 2013). Extrapolated values are only shown up to double the reference sampling size to avoid large prediction errors

At 95% sampling coverage, the three sampling methods were similarly effective as they did not reach statistically distinguishable mean species richness. Ultrasound recording had the lowest richness estimates, while mist‐netting tended to reach higher estimates than point counts with incidence‐based data, and lower ones with abundance‐based data (Figure 3). At low numbers of sampling hours (≲2.5) and of sampled individuals (≲20), bat point counts and automated recordings allowed to detect significantly more species than mist‐netting. Point counts still reached similar mean species richness when rarefaction‐extrapolation sampling curves used less conservative abundance estimates for point counts and ultrasound recordings (sum of detections), and including nonthermal detections raised estimated species richness above that of the other methods (Data S2, see previous comment).

### 3.3 Acoustic and thermal detection spaces

**FIGURE 4:**
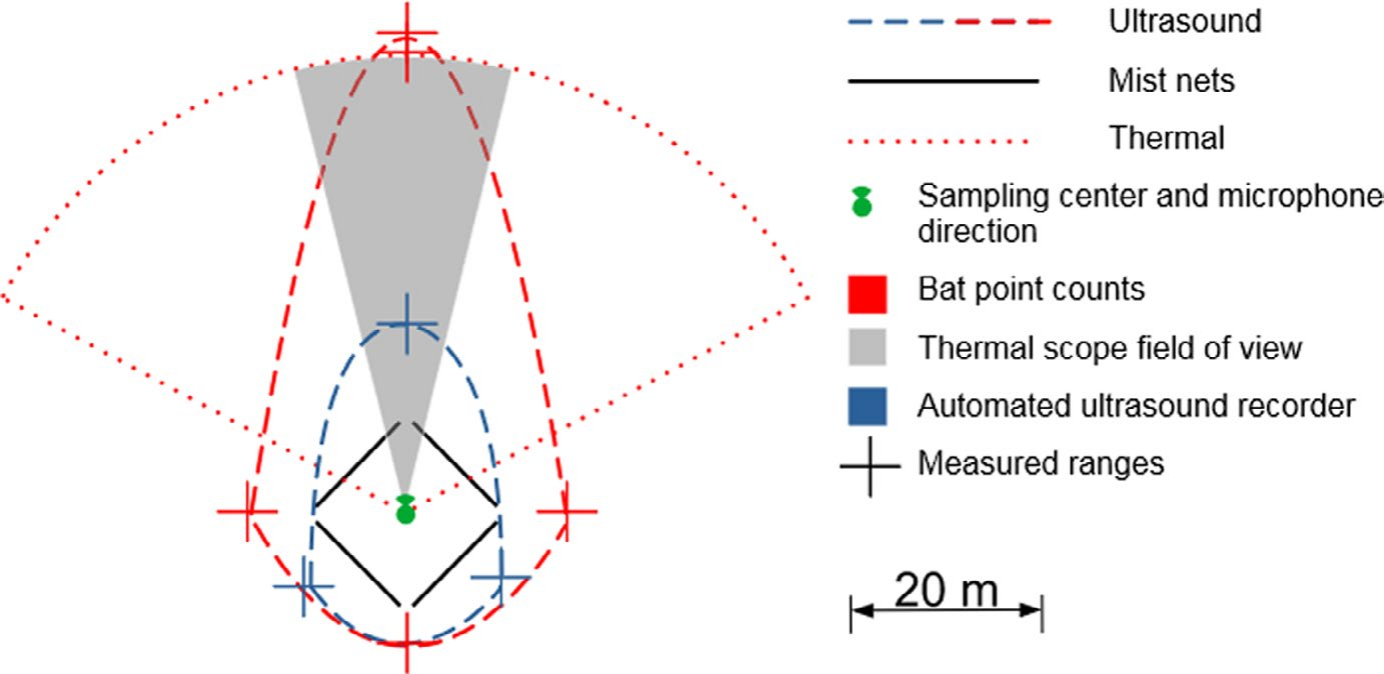
Detection ranges and sampling locations for bat point counts, mist nets, and automated ultrasound recorders. The sampling rig and ultrasound recorder were set up at the sampling center. The thermal scope's field of view was scanning the thermal detection area (red dotted line). The curved ranges for ultrasound were drawn manually between the measured range directions. The ultrasound detection ranges are scaled to a maximum of 50 m as they are only representative of our 40 kHz ultrasound emitter otherwise (SPL 48 dB @ 30 cm)

Bat point counts swept a large thermal detection area that encompassed a larger area than our mist nets, and their ultrasound detection spaces were larger and more directional than those of the automated ultrasound recorders (Figure 4). Ultrasound detection ranges of bat point counts (where the microphone was fitted with a horn) were almost three times larger than the unattended ultrasound recorders’ ranges (without horn) in the direction the microphone was pointing to (bat point counts: 450 m; automated ultrasound recorders: 164 m), and to some degree also to the side. The thermal scope had a range of 48 m on average, with a minimum of 19 m to a maximum of 84 m; its range was usually limited by obstacles such as oil palms or terrain irregularities. The mist nets approximately delimited an area of 150 m^2^ when a quadrilateral was drawn across their outer corners.

## 4 DISCUSSION

### 4.1 Bat point counts versus traditional sampling methods

For a sufficient sampling effort (i.e., >2.5 sampling hours or >20 individuals), all methods had a similar probability to detect new species and reached similar richness levels, even though they detected different species. In our study, mist‐netting tended to be less time‐ and workload‐efficient than point counts due to the hyper‐dominance of *Cynopterus brachyotis*. Passive acoustic monitoring tended to reach lower species richness levels as it cannot detect the five non‐echolocating bat species. Logistical constraints did not allow for longer sampling durations in bat point counts to detect more species and increase confidence of our estimates, and additional personnel could have introduced a sampling bias. Nonetheless, after one quarter of the sampling duration of the other sampling methods, bat point counts reached similar raw taxa counts. The workload (in terms of sampled individuals) efficiency of bat point counts compared with the other methods is currently statistically inconclusive; their performance must be evaluated in other regions and habitats. However, given that the bat point counts sample was almost complete in the shortest time—at which point it tended to detect more species than the other methods—the new method can be considered similarly effective but most time‐efficient in our study context. Bat point counts could be especially attractive for rapid assessments or for researchers with time constraints in the field.

Theoretically, passive collection of complementary acoustic and visual data enables bat point counts to detect echolocating and non‐echolocating bats without bias. Correspondingly, point counts could be particularly useful as a single sampling method in the Paleotropics and Oceania, where both types coexist. In our study, point counts reached high species richness at equal sampling coverage with the traditional sampling methods. Admittedly, if the aim was to detect a maximum number of species, combining mist nets with passive recording could yield better results than bat point counts. However, the logistical effort and expertise requirements should not be underestimated, and bat incidences from studies based on these vastly different methods are not directly comparable. As a result, bat point counts are currently the only way to obtain unbiased prevalences of echolocating and non‐echolocating bats with one method, foregoing potential methodological and taxonomic incompatibilities between sampling methods.

By design, bat point counts should have a smaller taxonomic sampling bias compared with established sampling methods. All flying bats must be detectable thermally as they are metabolically active, hence warmer than the surrounding environment. Their detectability depends mainly on their size and distance to the thermal scope. The larger bats we caught were approximately 10 cm large (from head to tail—roughly the palm of the hand used to determine thermal detection ranges), and could be detected—albeit presumably not identified—at up to 80 m. Geometrically, 4‐cm large bats would thus have a predictable detection area of 32 m radius. In contrast, bat diversity studies that are based on trapping and acoustics rarely account for the detectability of different species when comparing communities. Ultrasound detection ranges are highly variable and species‐specific as they depend strongly on the frequency, sound level, and directivity of the calls (Darras et al., 2016). Also mist‐netting species abundances depend on their exact setup, which cannot systematically be reproduced across studies, and given the taxonomic sampling biases mentioned earlier, they are unlikely to be comparable across species. It follows that with our approach, specific thermal detection ranges should be relatively easily measured and likely have less biased detectability between taxa, so that the corresponding density estimates could be computed more accurately and compared across species.

Bat point counts revealed that in oil palm plantations in our study region, there is a strong sampling bias against insectivorous bats. Acoustic monitoring alone cannot reveal the magnitude of that bias, and although mist‐netting potentially could, four echolocating species were not caught with our mist nets at all—even though Hipposideridae and Rhinolophidae were caught in other studies (Fukuda et al., 2009; Huang et al., 2014). Previous studies from oil palm plantations in South‐east Asia used only mist nets so far, and perhaps as a consequence, it is recurrently stated that they are dominated by frugivorous bats—especially *Cynopterus brachyotis* (Azhar et al., 2015; Fukuda et al., 2009; Mohd‐Azlan, 2019; Syafiq et al., 2016). In contrast, in our bat point counts, only seven out of 100 detections came from Pteropodidae, and this ratio might even be lower when considering their higher detectability due to their larger size. These results are consistent with the fact that oil palm monocultures do not provide food for Pteropodidae: In our study site, they usually fly through to forage on fig trees on river banks (pers. obs. Kevin Felix Arno Darras K.F.A.D., Ellena Yusti E.Y.). Thus, it appears that much of the bat assemblage is ignored when using only mist nets, underlining their strong taxonomic bias in our system (Huang et al., 2019), and suggesting similar biases in other tropical studies might exist.

The three methods we tested have different practical requirements (Table 1). Taxonomic expertise is needed for all methods, but in the case of point counts and automated ultrasound recordings, it can be delayed or out‐sourced: species identification can be done on computers, anytime. Data processing is time‐effective for mist‐netting, as data can typically be transferred from field sheets directly after or even during the survey. In contrast, sound recordings must usually be retrieved, uploaded (optionally), and annotated, and, for bat point counts, photographs must be processed analogously. In our study with our iteratively developed workflow, we estimate a post‐survey acoustic workload that is approximately equal to the duration of the acoustic recordings to annotate and identify bat passes. For bat point counts, another 2–5 min processing time per detection can be added to obtain detection statistics and identification features, however, the acoustic processing can be considerably shortened by focusing on thermal detections only, which make up only 6% of the total recording time in our case. Regarding initial costs, mist‐netting requires considerable training, and point counts currently require high expertise and expenses for the assembly of the sampling rig. In our study, high‐end hardware was already available, but we estimated lower costs for all methods with alternative hardware at similar performance (Table 1): much cheaper, equivalent sound recorders can be obtained (Hill et al., 2019), and to date, a complete rig can be built for approximately 2200 EUR. Although the initial hardware costs of bat point counts can be prohibitive for less well‐funded research projects, we strive to develop the bat point counts rig further (Darras et al., 2021) to lower the costs and increase accessibility. Finally, in comparison to mist‐netting, sound recorder installation and bat point counts can be carried out more easily by trained personnel, even alone, if safety at night is no concern.

**TABLE 1:** Comparison of practical aspects for the three bat sampling methods in our study, per sampling site and night, for comparable sampling effort and area

Method	Point counts	Mist‐netting (48 m × 3 m)	Automated ultrasound recording
Team size (persons)	1–2	2	1–2
Equipment bulk	Moderate	High	Small
Price per site (EUR)	2200 (current) to 3000 (this study)	100 to 800 (this study)	120 (current) to 1200 (this study)
Postsurvey data processing time	High	None	High
Expertise	Moderate (sampling) High (postprocessing)	High	Low (sampling) High (postprocessing)
Setup effort	Very high (initial assembly) Low (per survey)	High	Low
Sampling multiple sites	Not possible when continuously sampling with one team	Possible to sample 2 nearby sites continuously with one team	Possible to sample multiple sites simultaneously with several recorders

### 4.2 Application challenges and research opportunities

Point counts potentially cover large sampling areas, but they depend on the surveyed site, as clear lines of sight are required. In our study, the sparse understory allowed us to detect bats at relatively long ranges (48 m on average) that were only limited by larger obstacles such as palms or uneven terrain. We can predict that in even, open terrain without trees, the detection spaces would be even greater (approximately 80 m —the maximum range we measured). However, in previous trials with a prototype sampling rig in a forest with dense understory vegetation, the detection range was more limited (approximately 18 m, pers. obs. Ellena Yusti, E.Y.). Hence, we suggest choosing good vantage points or clearing lower, nearby vegetation that considerably obstructs the field of view. More importantly, irrespective of the variability of detection ranges across sampling sites, detection ranges are ultimately measurable so that detectability variations—especially across species—can be accounted for to obtain rough density estimates, an approach that is still rare but essential for mist‐ netting and acoustic studies (Meyer et al., 2011). Theoretically, bat point counts could even be used to derive more reliable density estimates from distance sampling approaches: using the first detection of each species (standard approach in distance sampling to avoid double‐counting) and its angular size, detection distances could be estimated. Finally, to solve the issue that only a part of the surroundings is thermally sampled at any point in time, thermal scopes with higher resolution and larger field of view could be used to cover a larger detection area.

Bat point counts are a fundamentally different method that requires human presence but does not capture any live specimens. Instead, users must become proficient with the handling of acoustic and photographic data. Our technical information and workflow (Darras et al., 2021) as well as our ecoacoustic software tool (Darras et al., 2020) and our identification key presented here facilitates that process. However, different sampling regions—and to some degree, habitats—require dedicated identification keys. So far, identification keys are based on absolute external, and sometimes internal morphological measurements and features, and rarely use acoustic data to our knowledge. Moreover, researchers often cannot make complementary ultrasound recordings when capturing bats, and flying‐tent or hand‐release recordings can yield calls that are atypical of free‐flying bats (Dietz & Kiefer, 2015). However, we showed that it is precisely the complementarity of acoustic and photographic data that could improve taxonomic identification, and we suggest that developing identification keys that combine acoustic and morphological features becomes a research priority.

Bat point counts will resolve more species and individuals as the technology matures and yields better near‐infrared imagery (Darras et al., 2021). One of our sonotypes remains unidentified, but it is possible that we could assign further detections to potential species with better images. Some detections included in our unresolved sonotype likely belonged to *Miniopterus* sp. based on the spectro‐temporal features of the sound, that is, the FM‐QCF calls with end frequency at 40–55 kHz (Joe Chuan‐Chia Huang, J.C.C.H. Huang et al., 2015). Nevertheless, proper species assignments are impossible unless more comprehensive reference call libraries become available for South‐east Asian bats. Likewise, frugivores are currently lumped into the *Cynopterus* genus because species identification requires internal and absolute body metrics. Still, it is possible to distinguish them from genera which have distinct head shapes and body sizes. Better near‐infrared imagery will increase the proportion of usable pictures, and reveal the wing bones and face more clearly. This would further improve the shape of the rarefaction‐extrapolation sampling curves and increase their confidence. Interestingly, we also photographed detailed morphological features that could aid in discriminating between individuals: we sexed a male *Cynopterus* by its visible penis, and identified it as an individual with a hole in its wing (Data S3). These individual signatures could yield more realistic abundance estimates and provide information for the conservation of wild populations.

Morphological and acoustic data from point counts could be invaluable for resolving species complexes (consisting of several candidate identifications). Analogously to our present attempts, we are hopeful that sonotypes can be resolved with more taxonomic depth, such as for “whispering” bat species (e.g., Phyllostomidae) (Yoh et al., 2020). The recordings obtained from bat point counts, using an ultrasonic horn, were also more amenable to call libraries: (1) their signal‐to‐noise ratio is consistently higher, as the horn amplifies the sounds from an actively tracked bat; (2) call durations are more accurately measurable, as echoes from the surroundings are shielded by the horn; (3) calls are representative of free‐flying bats, unlike calls from handheld bats, bats flying in tents, and likely also distraught, released bats. It follows that bat point counts can yield reference data to identify unresolved sonotypes inside unattended recordings, even *a posteriori*. Conversely, acoustic data can resolve “cryptic” species complexes that are morphologically almost indistinguishable: although we found only one member of the *Hipposideros bicolor* complex, *H. kunzi* was readily identified using its calls’ higher maximum energy frequency (Murray et al., 2018). Potentially, mist‐net captures could also be used to generate near‐infrared reference photographs for facilitating identification from pictures of free‐flying bats, using coloration differences that we were not able to use here. Finally, the flight pattern observed in the thermal scope can also be diagnostic (Darras, 2020), but its usefulness must be evaluated more thoroughly.

The combination of direct visual observation with ultrasonic recording allows studies on bat behavior (cf. pictures and animated GIFs in Data S3). Interactions between individuals (within and between species) can be observed—we saw several encounters between bats. In some cases, the bats would fly together, and in other cases, they would avoid each other. Possibly, the function of social calls could be elucidated and linked to competition for critical resources (Corcoran & Conner, 2014) or partners (Voigt et al., 2008), or calls from individual bats with different ages, sexes, and group memberships (Kao et al., 2020; Pfalzer & Kusch, 2003; Siemers et al., 2005). Moreover, flight maneuvers such as diving can be seen, giving insights about hunting behavior: we observed several dives and potentially a catch on the wing. Also, the head position was variable, appearing to indicate the echolocating direction for scanning prey and obstacles, and helping their identification. Lastly, the exact coupling of photographic and audio data reveals what calls are emitted in which situation or environment—for instance during a diving maneuver—and when exactly bats emit feeding buzzes and social calls (Middleton et al., 2014). Previously, such observations were only possible in carefully controlled artificial environments such as tunnels with extensive setups (Clark, 2021).

## 5 CONCLUSION

Bat point counts are a new tool for ecologists and a promising avenue for sampling flying bat communities comprehensively and efficiently. Technological advances will lower the cost and increase the practicability and efficacy of bat point counts in the near future (Darras et al., 2021). The method still needs to be evaluated further in different environments with more speciose bat assemblages. Obviously, mist‐netting continues to be needed for capturing and measuring undescribed species, for taking samples, and for assessing the physiology; it currently delivers the highest, usually species‐level identification accuracy. Also, automated ultrasound recordings remain an efficient, standardized, and practical way of sampling echolocating bats. Yet, bat point counts have unique advantages. For instance, they could be used with bird point counts to comprehensively and consistently sample all flying vertebrates, and they are the only zoonotically safe, direct observation method which could be used to engage the public in bat conservation (Rocha et al., 2020). The potential of newer technologies should be embraced to advance chiropterology and advance fundamental and applied research questions in ecology and conservation. Bat point counts, as a direct observation method that makes bats audible and visible, shine a new light on flying bat communities and their behavior, and will potentially lead to new insights.

## CONFLICT OF INTEREST

The authors declare to have no conflicts of interest.

## AUTHOR CONTRIBUTIONS

**Kevin Felix Arno Darras:** Conceptualization (lead); Data curation (equal); Formal analysis (lead); Funding acquisition (equal); Investigation (lead); Methodology (lead); Project administration (equal); Resources (lead); Software (lead); Supervision (lead); Validation (equal); Visualization (lead); Writing‐original draft (lead); Writing‐review & editing (lead). **Ellena Yusti:** Formal analysis (equal); Investigation (equal); Methodology (equal); Validation (equal); Writing‐review & editing (equal). **Joe Chun‐Chia Huang:** Formal analysis (equal); Investigation (equal); Resources (equal); Supervision (equal); Validation (equal); Writing‐review & editing (equal). **Delphine‐Clara Zemp:** Funding acquisition (equal); Investigation (equal); Supervision (equal); Validation (equal); Writing‐review & editing (equal). **Agus Priyono Kartono:** Funding acquisition (equal); Investigation (equal); Project administration (equal); Supervision (equal); Validation (equal). **Thomas Cherico Wanger:** Investigation (equal); Supervision (equal); Validation (equal); Writing‐review & editing (equal).

### OPEN RESEARCH BADGES

This article has been awarded <Open Materials, Open Data> Badges. All materials and data are publicly accessible via the Open Science Framework at: Sound recordings are on BioSounds (citation in text): https://soundefforts.uni‐goettingen.de/biosounds/collection/show/34; Infrared pictures are on OSF: https://osf.io/rqyh8/; Materials needed for research are cited and separately published in open access article: https://f1000research.com/articles/10‐189.

## Supporting information

Supplementary Material

Data S1

Data S2

Data S3

Supplementary Material

Supplementary Material

Supplementary Material

Supplementary Material

Supplementary Material

Supplementary Material

Supplementary Material

## Data Availability

The data required for reproducing our results are available in the supplementary materials. The technical details for assembling a bat point count rig, as well as the working protocol, are published elsewhere (Darras et al., 2021). The annotated ultrasound recordings are available publicly on BioSounds (Darras et al., 2020), and all infrared photographs as well as the sound transmission recordings are available on the Open Science Framework website (Darras, 2020).
