# Supplementary material for: Bat point counts: A novel sampling method shines light on flying bat communities
Source: Ecol Evol. 2021 Nov 30;11(23):17179–90. doi: 10.1002/ece3.8356 (PMC8668732; doi:10.1002/ece3.8356)
Supplement: Supplementary file 1 — Supplementary Material [file ECE3-11-17179-s006.docx]

## Supplementary Tables

**Table S1:** Bat echolocation call parameters for all detected echolocating species and sonotypes. Values were measured from bat point counts recordings, except for those species denoted with an asterisk, which were only detected in automated ultrasound recordings. Three bat calls were measured for each detection.

| **Bat call type or species** | **Call shape** | **Peak frequency (kHz)** | **Start frequency (kHz)** | **End Frequency (kHz)** | **Call duration (ms)** | **Inter-pulse**  **interval (ms)** | **Detections** |
| --- | --- | --- | --- | --- | --- | --- | --- |
| C (sonotype) | FM-QCF | 55 +/-3 | 86 +/-10 | 51 +/-3 | 10 +/-2 | 92 +/-20 | 39 |
| D (sonotype) | BFM | 55 +/-NA | 90 +/-NA | 52 +/-NA | 8 +/-NA | 64 +/-NA | 1 |
| Hipposideros kunzi * | CF | 142 +/-NA | 141 +/-NA | 117 +/-NA | 8 +/-NA | 17 +/-NA | 1 |
| Hipposideros orbiculus | CF | 80 +/-NA | 78 +/-NA | 70 +/-NA | 14 +/-NA | 48 +/-NA | 1 |
| Kerivoula sp. * | BFM | 98 +/-NA | 142 +/-NA | 64 +/-NA | 5 +/-NA | 57 +/-NA | 1 |
| Pipistrellus stenopterus | FM-QCF | 33 +/-1 | 47 +/-21 | 34 +/-6 | 14 +/-2 | 182 +/-79 | 7 |
| Rhinolophus sedulus | CF | 65 +/-1 | 61 +/-4 | 61 +/-4 | 43 +/-9 | 80 +/-11 | 3 |
| Scotophilus kuhlii | FM-QCF | 43 +/-6 | 74 +/-15 | 37 +/-4 | 12 +/-4 | 139 +/-60 | 20 |

##

## Supplementary Figures


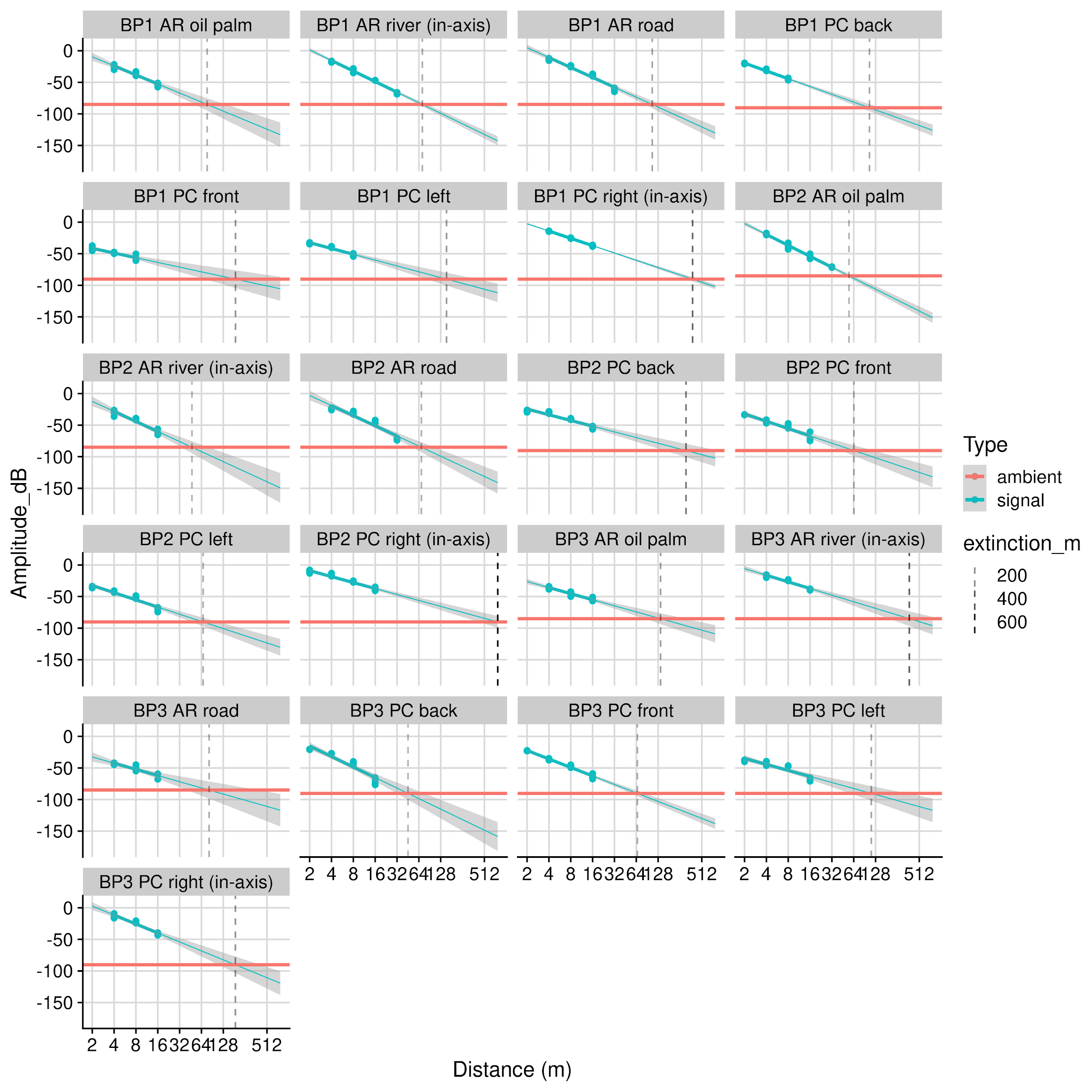


**Figure S1:** Ultrasound transmission profiles with distance for the different sampling sites, directions, and sampling methods (AR = acoustic recording, PC = point count). The red line depicts the mean ambient sound level per method, and the blue line shows a linear fit of log-transformed to the ultrasound test tone sound level. The intersection between both lines corresponds to the extinction distance for our particular test sound (@40 kHz, Ultrasound Calibrator, Wildlife Acoustics).


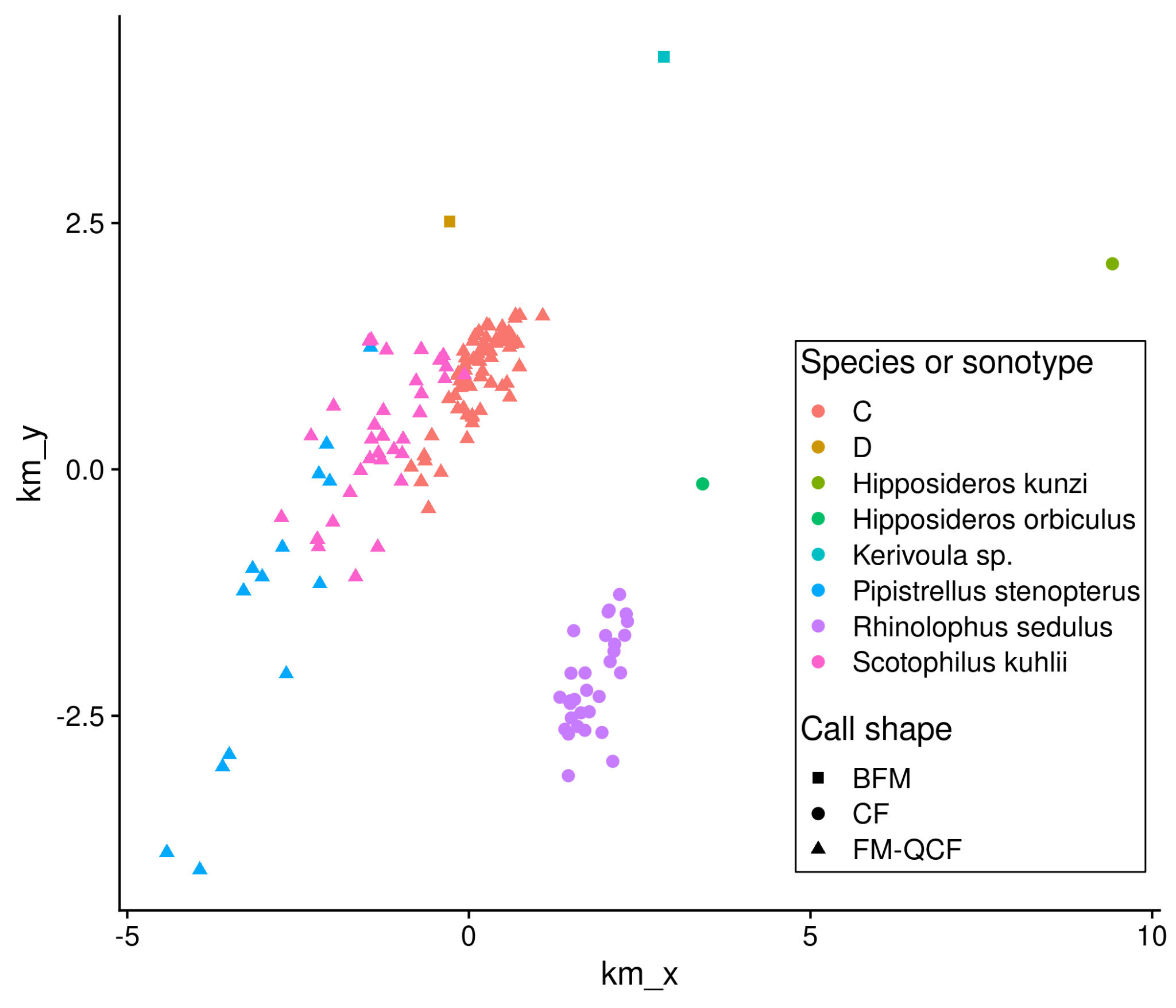


**Figure S2:** K-means clustering visualisation of 151 measured bat calls, both from automated ultrasound recordings and bat point count recordings. We used clearly recorded bat calls to collect numeric measurements of peak frequency, start and end frequency, call duration, inter-call interval, and call shape, which was coded as 0 for FM calls, 0.5 for FM-QCF calls, and 1 for CF calls.
