# Supplementary material for: Bat point counts: A novel sampling method shines light on flying bat communities
Source: Ecol Evol. 2021 Nov 30;11(23):17179–90. doi: 10.1002/ece3.8356 (PMC8668732; doi:10.1002/ece3.8356)
Supplement: Supplementary file 2 — Data S1 [file ECE3-11-17179-s003.pdf]

Analysis of bat point counts vs. mist-netting and automated ultrasound recording

Kevin F.A. Darras

2021-10-19

clear all variables

```
rm(list=ls())
```

loading packages

```
library(data.table)
library(ggplot2)
library(cowplot)
library(factoextra)
```

```
## Welcome! Want to learn more? See two factoextra-related books at https://goo.gl/ve
3WBa
```

```
library(iNEXT)
library(gridExtra)
library(seewave)
library(tuneR)
```

set working directory

```
setwd("/home/kdarras/Documents/Boulot/2020 Hangzhou/Bat point counts")
```

Prepare data

Import and merge data

```
tags0=fread("Data ecological/BioSounds tags_PC_AR.csv")
```

correcting Genus-level ID label

```
tags0[binomial=="Kerivoula",Species:="Kerivoula sp."]
mistnetting0=fread("Data ecological/Bats in 3 methods - Mist netting.csv")
```

note: BP1 Road replicate 1 US4_20190523_192936.flac: No bats detected; even though recordings can be longer, no detection data were entered after 10 min.

```
thermal0=fread("Data ecological/Bats in 3 methods - Thermal detections.csv")
surveys0=fread("Data ecological/Bats in 3 methods - Surveys.csv")
```

adapt data format

```
surveys0[,Date:=as.Date(Survey_date,format="%d/%m/%Y")]
plots0=fread("Data ecological/Bats in 3 methods - Plots.csv")
```

add survey info to thermal data

```
thermal1=merge(surveys0[Method=="Point count" & Point_count_recording!="",.N,.(Point_
count_recording,Number)]
               ,thermal0,by.x=c("Point_count_recording"),by.y=c("File_name"))
```

merge all tags with thermal data

```
tags1=merge(tags0,thermal1,by=c("thermal_ID","Plot"),all=T)
```

add survey night info to tags

```
tags2=merge(surveys0[,.N,.(Method,Date,Night)],tags1,by=c("Method","Date"))
```

Handle times

extract date for mist netting

```
mistnetting0[,Date:=as.Date(Date,format="%d/%m/%Y")]
```

exclude data from 27th (failed survey)

```
mistnetting1=mistnetting0[comments!="released" & Date!="2019-05-27"]
```

assign method

```
mistnetting1[,Method:="Mist-netting"]
```

add survey info

```
mistnetting2=merge(surveys0[Date!="2019-05-27",.N,.(Method,Date,Night)],mistnetting1,
by=c("Method","Date"))
```

converting times

```
tags2[,Date:=as.POSIXct(Date,format="%d/%m/%Y")]
```

extract exact time of recording

```
tags2[,Recording_time:=as.POSIXct(substr(recording.name,12,28),format="%Y%m%d_%H%M%S"
)]
```

exact time of detection

```
tags2[,Detection_start_time:=Recording_time+start_time]
tags2[,Detection_end_time:=Recording_time+end_time]
```

assign detection hours for ultrasound recording

```
tags2[Method=="Ultrasound recording",detection_hour:=as.numeric(format(Detection_star
t_time,format="%H"))-17]
```

a few detections are 1-2 minutes after 22:00, technically this is still within the fourth hour because sunset is a few minutes past 18:00

```
tags2[Method=="Ultrasound recording" & format(Detection_start_time,format="%H")==22,d
etection_hour:=4]
```

back-convert dates to format for future merge compatibility

```
tags2[,Date:=as.Date(Date,format="%d/%m/%Y")]
```

count cumulative hour of detection

```
tags2[Method=="Ultrasound recording",detection_hour_cumulative:=detection_hour+(Night
-1)*4]
tags2[Method=="Point count",detection_hour_cumulative:=Night]
```

compute exact capture times

```
mistnetting2[,capture_time:=as.POSIXct(paste(Date,Time_capture),format="%Y-%m-%d %H:%
M")]
```

calculate detection hours

```
mistnetting2[,detection_hour:=as.numeric(format(capture_time,format="%H"))-17]
```

back-convert dates format for future merge compatibility

```
mistnetting2[,Date:=as.Date(Date,format="%d/%m/%Y")]
```

count hour of detection cumulatively

```
mistnetting2[,detection_hour_cumulative:=detection_hour+(Night-1)*4]
```

Species identification

Sound and near-infrared images

count simultaneous tags/ultrasound detections for each thermal ID that has measured calls

```
for (ti in tags2[!is.na(thermal_ID) & !is.na(Photos_reference) & grepl("bat call",Spe
cies),tag_id]){

  start_time_temp=tags2[tag_id==ti,Detection_start_time]
  end_time_temp=tags2[tag_id==ti,Detection_end_time]

  simultaneous_tags_temp=nrow(tags2[Method=="Point count" & (Detection_end_time>start
_time_temp) & (Detection_start_time<end_time_temp) & tag_id!=ti])
  tags2[tag_id==ti,simultaneous_tags:=simultaneous_tags_temp]
}
```

table for checking calls and photos to iteratively help manual ID of sonotypes (excluding ambiguous detections with simultaneous bat calls)

```
tags2[!is.na(simultaneous_tags)
      ,.(`Sonotype`=Species,`thermal ID\n(GDrive picture folder)`=thermal_ID,`tag ID
\n(solid border tag)`=tag_id,link=paste("https://soundefforts.uni-goettingen.de/bioso
unds/recording/show/",recording_id,sep="")
         ,Detection_start_s=format(Detection_start_time,format="%H:%M:%S"),Frequency_
max_energy,Frequency_start,Frequency_end,Call_interval,Call_duration
         ,`Simultaneous acoustic detections\n(be careful if>0)`=simultaneous_tags,Eye
s,Ears,Tail_type,`FA/HB ratio`="",`FA/TL ratio`="",`TL/HB ratio`="",Checked_by_Joe=""
,Other_visible_features="")][order(Sonotype)]
```

```
##        Sonotype thermal ID\n(GDrive picture folder) tag ID\n(solid border tag)
##  1: A1 bat call                                   16                     33791
##  2: A1 bat call                                  536                     34567
##  3: B1 bat call                                   18                     23714
##  4: B1 bat call                                  405                     23508
##  5: B1 bat call                                  406                     35058
##  6: B1 bat call                                  407                     34739
##  7: B1 bat call                                  408                     35059
##  8: B1 bat call                                  412                     23972
##  9: B1 bat call                                  413                     23970
## 10: B1 bat call                                  415                     34730
## 11: B1 bat call                                  537                     35090
## 12: B1 bat call                                  546                     35098
## 13: C1 bat call                                   87                     24519
## 14: C1 bat call                                  104                     24529
## 15: C1 bat call                                  109                     24536
## 16: C1 bat call                                  111                     24540
## 17: C1 bat call                                  115                     33830
## 18: C1 bat call                                  165                     23946
## 19: C1 bat call                                  174                     23949
## 20: C1 bat call                                  257                     34570
## 21: C1 bat call                                  261                     34892
## 22: C1 bat call                                  262                     34895
## 23: C1 bat call                                  264                     34910
## 24: C1 bat call                                  269                     34909
## 25: C1 bat call                                  285                     34918
## 26: C1 bat call                                  306                     34259
## 27: C1 bat call                                  321                     24567
## 28: C1 bat call                                  337                     24599
## 29: C1 bat call                                  346                     35020
## 30: C1 bat call                                  421                     34094
## 31: C1 bat call                                  444                     24679
## 32: C1 bat call                                  449                     24685
## 33: C1 bat call                                  452                     23586
## 34: C1 bat call                                  601                     34947
## 35: C1 bat call                                  621                     24464
##        Sonotype thermal ID\n(GDrive picture folder) tag ID\n(solid border tag)
##                                                                    link
##  1: https://soundefforts.uni-goettingen.de/biosounds/recording/show/2954
##  2: https://soundefforts.uni-goettingen.de/biosounds/recording/show/2951
##  3: https://soundefforts.uni-goettingen.de/biosounds/recording/show/2954
##  4: https://soundefforts.uni-goettingen.de/biosounds/recording/show/2916
##  5: https://soundefforts.uni-goettingen.de/biosounds/recording/show/2916
##  6: https://soundefforts.uni-goettingen.de/biosounds/recording/show/2916
##  7: https://soundefforts.uni-goettingen.de/biosounds/recording/show/2916
##  8: https://soundefforts.uni-goettingen.de/biosounds/recording/show/2916
##  9: https://soundefforts.uni-goettingen.de/biosounds/recording/show/2916
## 10: https://soundefforts.uni-goettingen.de/biosounds/recording/show/2916
## 11: https://soundefforts.uni-goettingen.de/biosounds/recording/show/2951
## 12: https://soundefforts.uni-goettingen.de/biosounds/recording/show/2951
## 13: https://soundefforts.uni-goettingen.de/biosounds/recording/show/2979
## 14: https://soundefforts.uni-goettingen.de/biosounds/recording/show/2991
## 15: https://soundefforts.uni-goettingen.de/biosounds/recording/show/2980
## 16: https://soundefforts.uni-goettingen.de/biosounds/recording/show/2980
## 17: https://soundefforts.uni-goettingen.de/biosounds/recording/show/2986
## 18: https://soundefforts.uni-goettingen.de/biosounds/recording/show/2910
## 19: https://soundefforts.uni-goettingen.de/biosounds/recording/show/2911
```

```
## 20: https://soundefforts.uni-goettingen.de/biosounds/recording/show/2955
## 21: https://soundefforts.uni-goettingen.de/biosounds/recording/show/2955
## 22: https://soundefforts.uni-goettingen.de/biosounds/recording/show/2955
## 23: https://soundefforts.uni-goettingen.de/biosounds/recording/show/2955
## 24: https://soundefforts.uni-goettingen.de/biosounds/recording/show/2955
## 25: https://soundefforts.uni-goettingen.de/biosounds/recording/show/2960
## 26: https://soundefforts.uni-goettingen.de/biosounds/recording/show/2965
## 27: https://soundefforts.uni-goettingen.de/biosounds/recording/show/2993
## 28: https://soundefforts.uni-goettingen.de/biosounds/recording/show/2985
## 29: https://soundefforts.uni-goettingen.de/biosounds/recording/show/2981
## 30: https://soundefforts.uni-goettingen.de/biosounds/recording/show/2921
## 31: https://soundefforts.uni-goettingen.de/biosounds/recording/show/2978
## 32: https://soundefforts.uni-goettingen.de/biosounds/recording/show/2978
## 33: https://soundefforts.uni-goettingen.de/biosounds/recording/show/2923
## 34: https://soundefforts.uni-goettingen.de/biosounds/recording/show/2961
## 35: https://soundefforts.uni-goettingen.de/biosounds/recording/show/2956
##                                                                     link
##     Detection_start_s Frequency_max_energy Frequency_start Frequency_end
##  1:          19:41:12                34681           93140         33830
##  2:          19:00:11                   NA              NA            NA
##  3:          19:42:36                42454           91962         41863
##  4:          20:13:31                   NA              NA            NA
##  5:          20:14:06                   NA              NA            NA
##  6:          20:14:33                35319           75689         31385
##  7:          20:14:59                   NA              NA            NA
##  8:          20:17:07                   NA              NA            NA
##  9:          20:17:37                37321           92400         31041
## 10:          20:18:13                   NA              NA            NA
## 11:          19:00:09                   NA              NA            NA
## 12:          19:01:54                48716           72197         39641
## 13:          19:07:13                55839           78035         53446
## 14:          19:30:01                55759           73058         53885
## 15:          20:05:40                57504           93137         54945
## 16:          20:10:22                   NA              NA            NA
## 17:          20:27:29                55831           88707         53429
## 18:          20:34:23                56240           95440         53680
## 19:          20:47:25                59402           94927         49037
## 20:          20:03:25                   NA              NA            NA
## 21:          20:05:08                52389           82652         47371
## 22:          20:06:06                   NA              NA            NA
## 23:          20:08:35                   NA              NA            NA
## 24:          20:11:35                   NA              NA            NA
## 25:          20:23:04                55781           90094         51152
## 26:          20:38:03                52032           84241         47393
## 27:          19:26:33                55760           93920         52240
## 28:          19:40:29                   NA              NA            NA
## 29:          19:59:21                55080           93840         46081
## 30:          20:26:30                53090           84309         49059
## 31:          20:07:13                   NA              NA            NA
## 32:          20:11:50                54000           92160         46161
## 33:          19:08:20                55901           91690         53068
## 34:          19:37:34                48576           59689         46971
## 35:          19:53:50                56400           92720         50560
##     Detection_start_s Frequency_max_energy Frequency_start Frequency_end
##     Call_interval Call_duration
##  1:            90            10
##  2:            NA            NA
##  3:            90            10
```

```
##  4:            NA            NA
##  5:            NA            NA
##  6:           180            10
##  7:            NA            NA
##  8:            NA            NA
##  9:            87             8
## 10:            NA            NA
## 11:            NA            NA
## 12:            80            13
## 13:            63            13
## 14:           127            10
## 15:            80            11
## 16:            NA            NA
## 17:            69             8
## 18:           100            10
## 19:            81             9
## 20:            NA            NA
## 21:            94            13
## 22:            NA            NA
## 23:            NA            NA
## 24:            NA            NA
## 25:           110             7
## 26:            85             9
## 27:            75            10
## 28:            NA            NA
## 29:            81             8
## 30:            63             8
## 31:            NA            NA
## 32:           107             9
## 33:           112            10
## 34:           107            12
## 35:            85            10
##     Call_interval Call_duration
##     Simultaneous acoustic detections\n(be careful if>0)        Eyes        Ears
##  1:                                                    2       small       small
##  2:                                                    2 not visible not visible
##  3:                                                    0       small       small
##  4:                                                    0       small not visible
##  5:                                                    0       small not visible
##  6:                                                    0       small not visible
##  7:                                                    1       small not visible
##  8:                                                    0       small not visible
##  9:                                                    0       small not visible
## 10:                                                    0 not visible not visible
## 11:                                                    2 not visible not visible
## 12:                                                    4       small       small
## 13:                                                    0       small       small
## 14:                                                    0 not visible       small
## 15:                                                    0       small       small
## 16:                                                    0       small       small
## 17:                                                    0       small       small
## 18:                                                    0       small       small
## 19:                                                    1       small       small
## 20:                                                    3 not visible       small
## 21:                                                    0       small not visible
## 22:                                                    1       small       small
## 23:                                                    1       small       small
## 24:                                                    0       small       small
```

```
## 25:                                                   1 not visible not visible
## 26:                                                   0       small       small
## 27:                                                   1       small not visible
## 28:                                                   0       small       small
## 29:                                                   0       small not visible
## 30:                                                   0       small not visible
## 31:                                                   0       small       small
## 32:                                                   0       small       small
## 33:                                                   0       small       small
## 34:                                                   1       small       small
## 35:                                                   0       small       small
##     Simultaneous acoustic detections\n(be careful if>0)        Eyes        Ears
##       Tail_type FA/HB ratio FA/TL ratio TL/HB ratio Checked_by_Joe
##  1:           B
##  2:           B
##  3:           B
##  4:           B
##  5:           B
##  6: not visible
##  7:           B
##  8:           B
##  9:           B
## 10:           B
## 11:           B
## 12:           B
## 13:           B
## 14: not visible
## 15: not visible
## 16:           B
## 17:           B
## 18:           B
## 19:           B
## 20:           B
## 21:           B
## 22:           B
## 23:           B
## 24:           B
## 25:           B
## 26:           B
## 27: not visible
## 28:           B
## 29:           B
## 30: not visible
## 31:           B
## 32:           B
## 33:           B
## 34:           B
## 35:           B
##       Tail_type FA/HB ratio FA/TL ratio TL/HB ratio Checked_by_Joe
##     Other_visible_features
##  1:
##  2:
##  3:
##  4:
##  5:
##  6:
##  7:
##  8:
```

```
##  9:
## 10:
## 11:
## 12:
## 13:
## 14:
## 15:
## 16:
## 17:
## 18:
## 19:
## 20:
## 21:
## 22:
## 23:
## 24:
## 25:
## 26:
## 27:
## 28:
## 29:
## 30:
## 31:
## 32:
## 33:
## 34:
## 35:
##     Other_visible_features
```

exclude inconclusive detections (no photos, no ultrasound) and night with incomplete surveys

```
tags3=tags2[!(Photos_with_bat==0 & Species=="thermal bat detection") & Date!="2019-05
-27"]
```

define Pteropodids: thermally detected (with photos) but no ultrasound or clearly visible morphology

```
tags3[Eyes=="large" | Tail_type=="A" | (Eyes=="not visible" & Ears=="not visible" & T
ail_type=="not visible" & binomial=="thermal bat detection")
      ,Species:="PTEROPODIDAE"]
```

check inconclusive thermal bat detections with pictures this one did not have ultrasound but clearly looked like Rhinolophus/Hipposideros

```
tags3[Species=="thermal bat detection"]
```

```
##         Method       Date Night N.x thermal_ID Plot
## 1: Point count 2019-05-23     1   6        119  BP1
##                      recording.name               Species tag_id recording_id
## 1: BP1_PC_US4_20190523_191742.flac thermal bat detection  23106         2912
##    start_time end_time activity_s Frequency_max_energy Frequency_start
## 1:        419   425.25       6.25                   NA              NA
##    Frequency_end Call_interval Call_duration comments             binomial
## 1:            NA            NA            NA   ID:119 thermal bat detection
##    min_freq max_freq number_of_individuals    Point_count_recording Number N.y
## 1:        1     5000                     1 US4_20190523_191742.flac      2   1
##                                                              link_BioSounds
## 1: https://soundefforts.uni-goettingen.de/biosounds/recording/show/2912
##    Flight_mention Echolocation_calls_simultaneous Photos_with_bat
## 1:     no mention                               0               3
##              Photos_reference  Eyes  Ears Tail_type
## 1: P1070936,P1070938,P1070939 small small         B
##                               Other_features_photo Comments      Recording_time
## 1: Rhinolophus/Hipposideros from tail type and ear          2019-05-23 19:17:42
##    Detection_start_time  Detection_end_time detection_hour
## 1:  2019-05-23 19:24:41 2019-05-23 19:24:47             NA
##    detection_hour_cumulative simultaneous_tags
## 1:                         1                NA
```

```
tags3[Species=="thermal bat detection",Species:="Rhinolophus/Hipposideros sp."]
```

remove detection that cannot be distinguished from either family

```
tags3=tags3[!Species=="Rhinolophus/Hipposideros sp."]
```

assign identity to A and B sonotypes

```
tags3[Species=="A1 bat call",Species:="Pipistrellus stenopterus"]
tags3[Species=="B1 bat call",Species:="Scotophilus kuhlii"]
```

optional assignment of probable IDS (does not affect SAC results)

```
# tags3[Species=="PTEROPODIDAE",Species:="Cynopterus sp."]
# mistnetting2[Species=="Kerivoula pellucida",Species:="Kerivoula"]
```

Sonotypes on K-means graph

Rhinolophidae and Hipposideridae have CF calls

```
tags3[grepl("Hipposideros",Species) | grepl("Rhinolophus",Species),call_shape:="CF"]
```

assign BFM calls

```
tags3[Species %in% c("Kerivoula sp.","D1 bat call"),call_shape:="BFM"]
```

set others as FM-QCF calls if not a pure thermal detection

```
tags3[is.na(call_shape) & Species!="thermal bat detection",call_shape:="FM-QCF"]
```

convert call type to numerical for use with K-means

```
tags3[call_shape=="FM-QCF",call_shape_int:=0.5]
tags3[call_shape=="BFM",call_shape_int:=0]
tags3[call_shape=="CF",call_shape_int:=1]
```

choose tags with measured calls for K-means, exclude G1 social calls calculate the inter-pulse interval as it was measured purely as the interval duration between calls

```
tags.measured0=tags3[!is.na(Frequency_max_energy) & !Species %in% c("G1 bat call")
                     ,.(tag_id,call_shape,call_shape_int,Species,Frequency_max_energ
y,Frequency_start,Frequency_end
                        ,Pulse_interval=Call_interval+Call_duration,Call_duration,Met
hod)]
```

k-means clustering

```
kmeans.tags=kmeans(tags.measured0[,-c("tag_id","Species","call_shape","Method")],8,ns
tart=25)
```

get coordinates for plotting

```
kmeans.coordinates=fviz_cluster(kmeans.tags,data=tags.measured0[,-c("tag_id","Specie
s","call_shape","Method")]
                                ,ellipse.type = "convex"
                                ,ggtheme = theme_minimal())
```

save coordinates into tag table

```
tag.km.coordinates=data.table(kmeans.coordinates$data)[,.(km_x=x,km_y=y,cluster)]
```

add total detections count

```
tags.measured1=merge(cbind(tags.measured0,tag.km.coordinates)
                     ,tags3[,.(detections_total=.N),.(Species)],by="Species")
```

draw K-means

```
ggplot(tags.measured1,aes(km_x,km_y,color=sub("1 bat call","",Species),shape=call_sha
pe))+
  # geom_text(aes(label=round(Frequency_max_energy/1000)),color="black",position = po
sition_nudge(y=-0.1))+
  # geom_text(data=tags.measured1[Species=="B1 bat call"],aes(label=tag_id),position
 = position_nudge(y=-0.1))+
  scale_shape_manual(values=c(15,19,17),name="Call shape")+
  scale_color_discrete(name="Species or sonotype")+
  geom_point(size=2)+
  theme_cowplot()+
  theme(legend.position=c(0.65,0.34),legend.box.background = element_rect(color="blac
k"),legend.box.margin = margin(t=2,r=2,b=2,l=2))
```

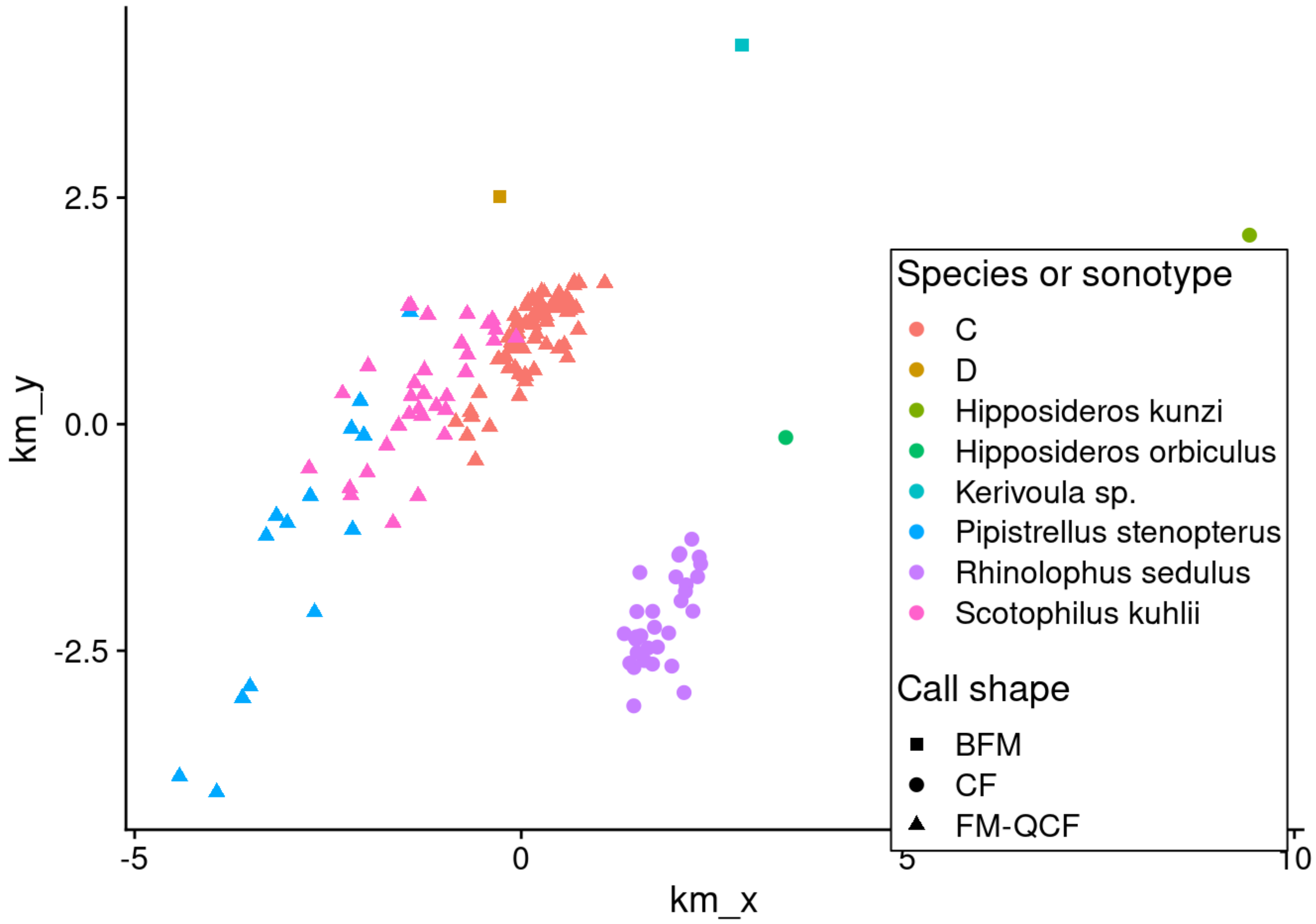


```
ggsave("Figures ecological/Fig S3.png",width=7,height=6)
ggsave("Figures ecological/Fig S3.svg",width=7,height=6)
```

compute summary statistics

```
TS1=tags.measured1[
  Method=="Point count" | Species %in% c("Hipposideros kunzi","Kerivoula sp.")
  ,.(`Call shape`=unique(call_shape)
     ,`Peak frequency (kHz)`=paste(round(mean(Frequency_max_energy/1000))," +/-",roun
d(sd(Frequency_max_energy)/1000),sep="")
     ,`Start frequency (kHz)`=paste(round(mean(Frequency_start)/1000)," +/-",round(sd
(Frequency_start)/1000),sep="")
     ,`End Frequency (kHz)`=paste(round(mean(Frequency_end)/1000)," +/-",round(sd(Fre
quency_end)/1000),sep="")
     ,`Call duration (ms)`=paste(round(mean(Call_duration))," +/-",round(sd(Call_dura
tion)),sep="")
     ,`Inter-pulse\ninterval (ms)`=paste(round(mean(Pulse_interval))," +/-",round(sd
(Pulse_interval)),sep="")
     ,Detections=.N)
  ,.(`Bat call type or species`=sub("1 bat call"," (sonotype)",Species))]
TS1[`Bat call type or species` %in% c("Hipposideros kunzi","Kerivoula sp.")
    ,`Bat call type or species`:=paste(`Bat call type or species`,"*")]
TS1
```

```
##    Bat call type or species Call shape Peak frequency (kHz)
## 1:             C (sonotype)     FM-QCF             55 +/-3
## 2:             D (sonotype)        BFM            55 +/-NA
## 3:     Hipposideros kunzi *         CF           142 +/-NA
## 4:   Hipposideros orbiculus         CF            80 +/-NA
## 5:          Kerivoula sp. *        BFM            98 +/-NA
## 6: Pipistrellus stenopterus     FM-QCF             33 +/-1
## 7:      Rhinolophus sedulus         CF             65 +/-1
## 8:       Scotophilus kuhlii     FM-QCF             43 +/-6
##    Start frequency (kHz) End Frequency (kHz) Call duration (ms)
## 1:             86 +/-10            51 +/-3            10 +/-2
## 2:             90 +/-NA           52 +/-NA            8 +/-NA
## 3:            141 +/-NA          117 +/-NA            8 +/-NA
## 4:             78 +/-NA           70 +/-NA           14 +/-NA
## 5:            142 +/-NA           64 +/-NA            5 +/-NA
## 6:             47 +/-21            34 +/-6            14 +/-2
## 7:              61 +/-4            61 +/-4            43 +/-9
## 8:             74 +/-15            37 +/-4            12 +/-4
##    Inter-pulse\ninterval (ms) Detections
## 1:                  92 +/-20         39
## 2:                  64 +/-NA          1
## 3:                  17 +/-NA          1
## 4:                  48 +/-NA          1
## 5:                  57 +/-NA          1
## 6:                 182 +/-79          7
## 7:                  80 +/-11          3
## 8:                 139 +/-60         20
```

```
fwrite(TS1,"Figures ecological/Table S1.csv")
```

Species lists

point count detections (thermal)

```
tags3[!is.na(thermal_ID),.(detections=.N,Photos_with_bat=sum(Photos_with_bat)),Specie
s]
```

```
##                     Species detections Photos_with_bat
## 1:              PTEROPODIDAE          7              66
## 2:               C1 bat call         54             629
## 3: Pipistrellus stenopterus         11              19
## 4:       Scotophilus kuhlii         26             195
## 5:   Hipposideros orbiculus          1               9
## 6:      Rhinolophus sedulus          1               0
```

point count detections (thermal and acoustic)

```
#put Rhinolophidae together
tags3[Species=="Rhinolophus sedulus" & Method=="Point count",Species:="RHINOLOPHIDAE"
]
tags3[Method=="Point count",.(detections=.N),Species]
```

```
##                      Species detections
## 1: Pipistrellus stenopterus        297
## 2:       Scotophilus kuhlii        341
## 3:              C1 bat call        239
## 4:             PTEROPODIDAE          7
## 5:   Hipposideros orbiculus          1
## 6:              D1 bat call          1
## 7:            RHINOLOPHIDAE         12
```

automated ultrasound recorders detections

```
tags3[Method=="Ultrasound recording",.(detections=.N),Species]
```

```
##                      Species detections
## 1: Pipistrellus stenopterus        771
## 2:       Scotophilus kuhlii        584
## 3:              C1 bat call        881
## 4:      Rhinolophus sedulus         29
## 5:            Kerivoula sp.          1
## 6:       Hipposideros kunzi          1
```

mist-netting captures

```
mistnetting2[,.(captures=.N),Species]
```

```
##                   Species captures
## 1: Cynopterus brachyotis       59
## 2:    Cynopterus minutus        4
## 3:  Macroglossus minimus        2
## 4:    Scotophilus kuhlii        7
## 5:   Kerivoula pellucida        2
## 6:     Cynopterus sphinx        2
## 7:           Myotis sp.1        1
```

Rarefaction-extrapolation sampling curves

Incidence-based curves (per hour)

merge tags and mist netting

```
species.night0=rbind(tags3[Method=="Ultrasound recording" |
                             (Method=="Point count" & !is.na(thermal_ID))
                           ,.(Species=unique(Species)),.(Method,detection_hour_cumula
tive)]
                     ,mistnetting2[,.(Species=unique(Species),Method="Mist-netting"
),.(detection_hour_cumulative)])
```

cast by species and night

```
species.night1=dcast(species.night0,Method+detection_hour_cumulative~Species,fun.aggr
egate=length)
```

split by method for iNEXT

```
species.night.pc=species.night1[Method=="Point count"]
species.night.ur=species.night1[Method=="Ultrasound recording"]
```

insert hours with no detections into mist netting data

```
species.night.mn0=merge(species.night1[Method=="Mist-netting"]
      ,data.table(Method="Mist-netting",detection_hour_cumulative=1:12)
      ,all.y=T)
```

replace NAs with zeroes

```
species.night.mn=cbind(species.night.mn0[,.(Method)],setnafill(species.night.mn0[,-"M
ethod"],fill=0))
```

put method-split data.tables in list for iNEXT

```
species.list=list(t(species.night.pc[,-c("Method","detection_hour_cumulative")])
                  ,t(species.night.ur[,-c("Method","detection_hour_cumulative")])
                  ,t(species.night.mn[,-c("Method","detection_hour_cumulative")]))
names(species.list)=c("Point count","Ultrasound recording","Mist-netting")
```

compute RE curves

```
incidence.curves=iNEXT(species.list,datatype = "incidence_raw",q=0,conf=0.83)
```

extract asymptotic richness

```
estimated.richness.incidence=data.table(estimateD(species.list,datatype="incidence_ra
w",base="coverage",level=0.95,conf=0.83))[order==0]
setnames(estimated.richness.incidence,c("t","site","qD"),c("Sampling hours","Method",
"richness"))
estimated.richness.incidence
```

```
##                  Method Sampling hours       method order    SC richness qD.LCL
## 1:          Point count              5 extrapolated     0 0.954    6.741  4.662
## 2: Ultrasound recording             11 interpolated     0 0.960    5.833  5.069
## 3:         Mist-netting             22 extrapolated     0 0.950    8.406  5.750
##    qD.UCL
## 1:  8.820
## 2:  6.598
## 3: 11.061
```

generate first graph

```
plot.i=ggiNEXT(incidence.curves)+
  labs(x="Sampling hours",y="Bat species richness")+
  geom_segment(data=estimated.richness.incidence,aes(x=`Sampling hours`,xend=`Samplin
g hours`,y=0,yend=richness,color=Method),lty=2)+
  geom_label(data=estimated.richness.incidence,aes(x=`Sampling hours`,label=round(ric
hness,1),y=richness,color=Method))+
  labs(subtitle = "Raw incidence-based")+
  theme_cowplot()+
  theme(legend.position="none")
```

Abundance-based curves

calculate conservative abundances from tags

```
tags.abundance=tags3[Method=="Ultrasound recording" |
                       (Method=="Point count" & !is.na(thermal_ID))
                     ,.(abundance.night.plot=max(number_of_individuals)),.(Method,Spe
cies,Night,Plot)]
```

merge tags and mist netting data

```
species.abundance0=rbind(tags.abundance[,.(abundance=sum(abundance.night.plot)),.(Met
hod,Species)]
                         ,mistnetting2[,.(abundance=.N),.(Method,Species)])
```

cast by species and method

```
species.abundance1=dcast(species.abundance0,Species~Method,value.var = "abundance")
species.abundance1[is.na(species.abundance1)]=0
species.abundance2=as.data.frame(species.abundance1[,.(`Point count`,`Mist-netting`,`
Ultrasound recording`)])
```

compute RE curves

```
abundance.curves=iNEXT(species.abundance2,datatype="abundance",q=0,conf=0.83
                       # ,endpoint=3
)
```

extract asymptotic richness

```
estimated.richness.abundance=data.table(estimateD(species.abundance2,datatype="abunda
nce",base="coverage",level=0.95,conf=0.83))[order==0]
setnames(estimated.richness.abundance,c("m","site","qD"),c("Sampled individuals","Met
hod","richness"))
estimated.richness.abundance
```

```
##                   Method Sampled individuals       method order    SC richness
## 1:          Point count                  31 extrapolated     0 0.952    6.307
## 2:          Mist-netting                 44 interpolated     0 0.950    5.998
## 3: Ultrasound recording                  20 interpolated     0 0.950    4.844
##    qD.LCL qD.UCL
## 1:  5.323  7.291
## 2:  4.968  7.028
## 3:  4.362  5.325
```

generate second graph

```
plot.a=ggiNEXT(abundance.curves)+
  labs(x="Sampled individuals",y="Bat species richness")+
  geom_segment(data=estimated.richness.abundance,aes(x=`Sampled individuals`,xend=`Sa
mpled individuals`,y=0,yend=richness,color=Method),lty=2)+
  geom_label(data=estimated.richness.abundance,aes(x=`Sampled individuals`,label=roun
d(richness,1),y=richness,color=Method),show.legend = FALSE)+
  labs(subtitle = "Abundance-based")+
  theme_cowplot()+
  theme(legend.position="bottom",legend.title = element_blank(),legend.box="vertical"
,legend.key.width = unit(2, 'cm'))
```

plot and save raw incidence and abundance-based RE curves

```
g=arrangeGrob(plot.i,plot.a,nrow=2)
plot(g)
```

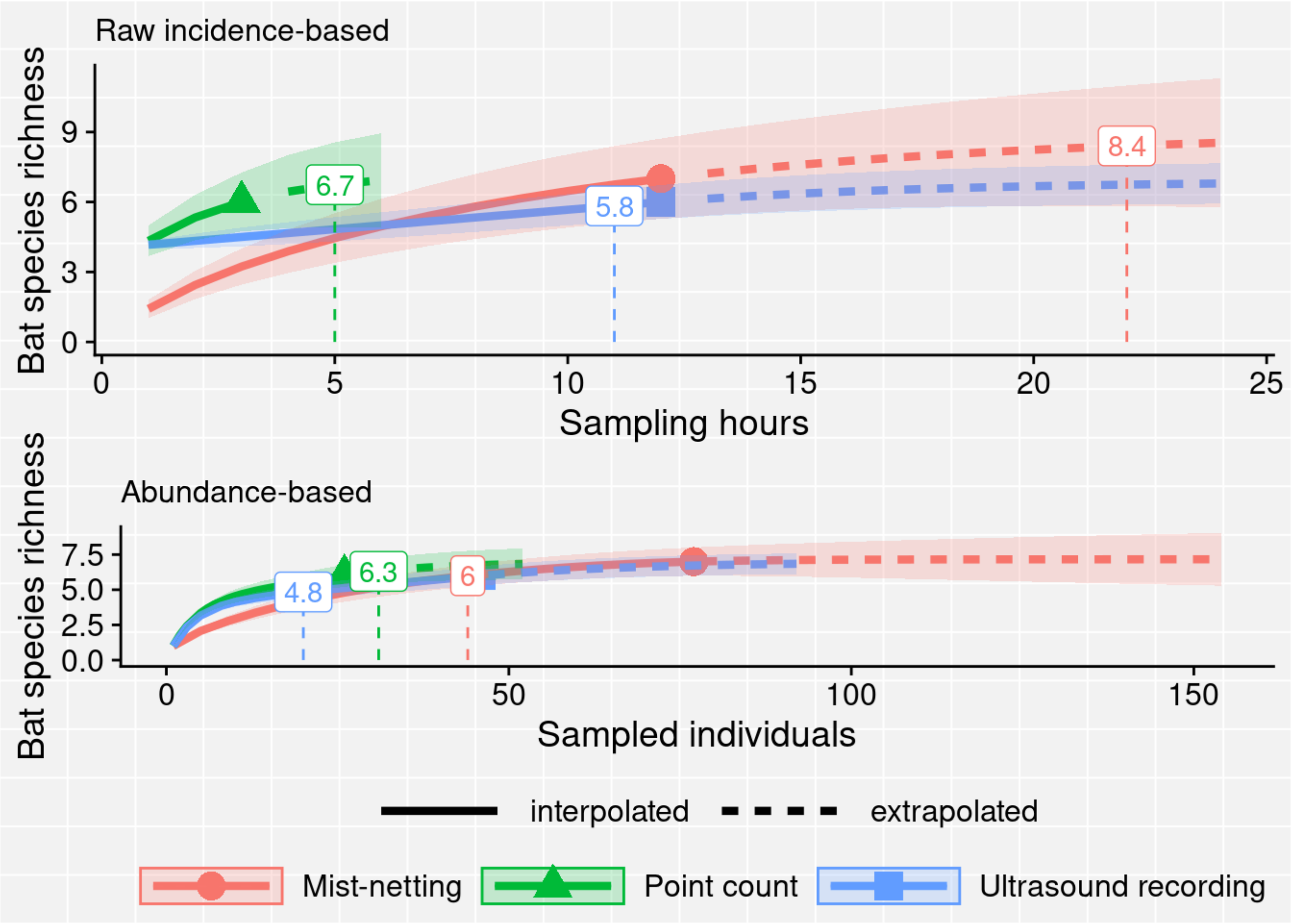

```
ggsave("Figures ecological/Fig 3.png",g,width=7,height=9)
ggsave("Figures ecological/Fig 3.svg",g,width=7,height=9)
```

Additional RE curves for supplement

Abundance-based RE curves with high-bound abundance estimates

use the sum instead of maximum to regard each detection as one different individual

```
tags.abundance.sum=tags3[Method=="Ultrasound recording" | (Method=="Point count" & !i
s.na(thermal_ID))
                         ,.(abundance.night.plot=sum(number_of_individuals)),.(Metho
d,Species,Night,Plot)]
```

including non-thermal detections for abundance

```
tags.abundance.nonthermal=tags3[Method=="Ultrasound recording" | (Method=="Point coun
t")
                                ,.(abundance.night.plot=max(number_of_individuals)),.
(Method,Species,Night,Plot)]
```

merge tags and mist netting data

```
species.abundance.sum0=rbind(tags.abundance.sum[,.(abundance=sum(abundance.night.plo
t)),.(Method,Species)]
                             ,mistnetting2[,.(abundance=.N),.(Method,Species)])
species.abundance.nonthermal0=rbind(tags.abundance.nonthermal[,.(abundance=sum(abunda
nce.night.plot)),.(Method,Species)]
                                    ,mistnetting2[,.(abundance=.N),.(Method,Specie
s)])
```

cast by species and method

```
species.abundance.sum1=dcast(species.abundance.sum0,Species~Method,value.var = "abund
ance")
species.abundance.sum1[is.na(species.abundance.sum1)]=0
species.abundance.sum2=as.data.frame(species.abundance.sum1[,.(`Point count`,`Mist-ne
tting`,`Ultrasound recording`)])
```

non-thermal data

```
species.abundance.nonthermal1=dcast(species.abundance.nonthermal0,Species~Method,valu
e.var = "abundance")
species.abundance.nonthermal1[is.na(species.abundance.nonthermal1)]=0
species.abundance.nonthermal2=as.data.frame(species.abundance.nonthermal1[,.(`Point c
ount`,`Mist-netting`,`Ultrasound recording`)])
```

compute RE curves

```
abundance.curves.sum=iNEXT(species.abundance.sum2,datatype="abundance",q=0,endpoint=2
500)
abundance.curves.nonthermal=iNEXT(species.abundance.nonthermal2,datatype="abundance",
q=0)
```

Raw incidence-based curves including non-thermal detections

```
species.night.nonthermal0=rbind(tags3[Method=="Ultrasound recording" | (Method=="Poin
t count")
                                      ,.(Species=unique(Species)),.(Method,detection_
hour_cumulative)]
                                ,mistnetting2[,.(Species=unique(Species),Method="Mist
-netting"),.(detection_hour_cumulative)])
```

cast by species and night

```
species.night.nonthermal1=dcast(species.night.nonthermal0,Method+detection_hour_cumul
ative~Species,fun.aggregate=length)
```

assign method

```
species.night.pc.nonthermal=species.night.nonthermal1[Method=="Point count"]
```

split methods again for iNEXT list

```
species.list.nonthermal=list(t(species.night.pc.nonthermal[,-c("Method","detection_ho
ur_cumulative")])
                             ,t(species.night.ur[,-c("Method","detection_hour_cumulat
ive")])
                             ,t(species.night.mn[,-c("Method","detection_hour_cumulat
ive")]))
names(species.list.nonthermal)=c("Point count","Ultrasound recording","Mist-netting")
```

compute RE curves

```
incidence.curves.nonthermal=iNEXT(species.list.nonthermal,datatype = "incidence_raw",
q=0)
```

plotting all curves for supplementary figure

```
plot.first=ggiNEXT(abundance.curves.sum)+labs(title="Abundance-based",subtitle="sum o
f all detections, only thermal detections")+theme_cowplot()+
  theme(legend.title = element_blank(),legend.key.width = unit(2, 'cm'))
plot1=ggiNEXT(incidence.curves.nonthermal)+labs(title="Raw incidence-based",subtitle=
"including non-thermal detections",x="sampling hours")+theme_cowplot()+theme(legend.p
osition="none")
plot2=ggiNEXT(abundance.curves.nonthermal)+labs(title="Abundance-based",subtitle="inc
luding non-thermal detections")+theme_cowplot()+theme(legend.position="none")
plot3=ggiNEXT(incidence.curves,type=3)+theme_cowplot()+theme(legend.position="none")+
labs(subtitle="only thermal detections")
plot4=ggiNEXT(abundance.curves,type=3)+theme_cowplot()+theme(legend.position="none")+
labs(subtitle="only thermal detections")
plot5=ggiNEXT(incidence.curves,type=2)+theme_cowplot()+theme(legend.position="none")+
labs(subtitle="only thermal detections",x="sampling hours")
plot6=ggiNEXT(abundance.curves,type=2)+theme_cowplot()+theme(legend.position="none")+
labs(subtitle="only thermal detections")

plot_grid(plot.first,arrangeGrob(plot1,plot2,plot3,plot4,plot5,plot6),rel_heights = c
(1,3),ncol=1)
```

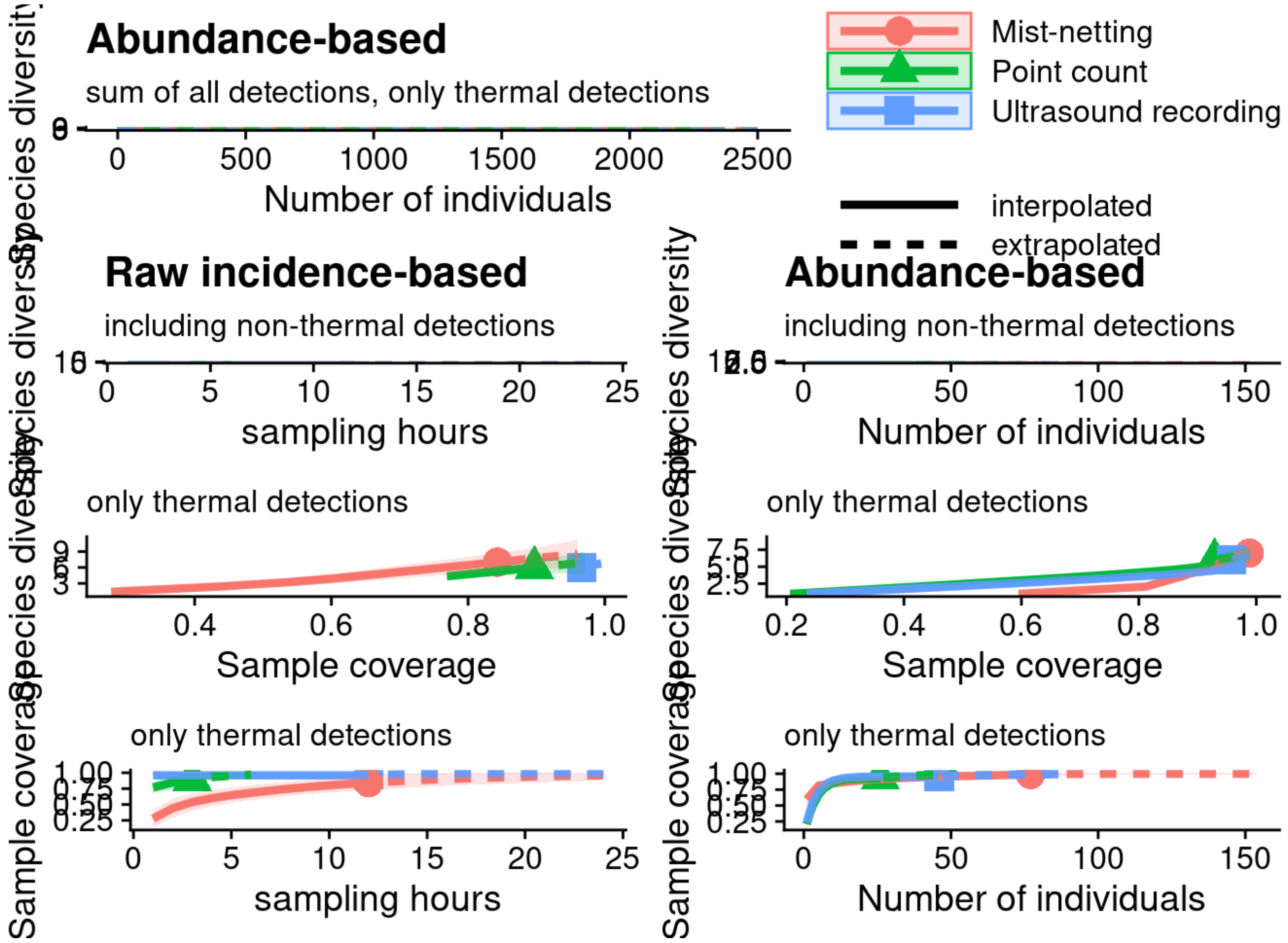


```
ggsave("Figures ecological/Fig S2.png",width=8,height=12)
ggsave("Figures ecological/Fig S2.svg",width=8,height=12)
```

Detection spaces

Sound

importing data pinpointing test sound temporal locations inside sound transmission files

```
ST0=fread("Data ecological/Bats in 3 methods - Sound transmission.csv")
ST0[,Plot:=substr(Filename,4,6)]
ST0[,Method:=substr(Filename,8,9)]
```

create empty column for amplitudes to measure

```
ST0[,Amplitude_dB:=numeric()]
```

denote directions for interpretation

```
ST0[Method=="AR" & Direction=="river",Direction:=paste(Direction,"(in-axis)")]
ST0[Method=="PC" & Direction=="right",Direction:=paste(Direction,"(in-axis)")]
```

define constants

```
frequency_bin=427
duration_signal_s=0.07
duration_ambient_s=60
```

Sound transmission recordings (available on https://osf.io/rqyh8/ (https://osf.io/rqyh8/)) must be in “Sound transmission” folder under the working directory importing sound transmission recording that contains ambient sound (to avoid different normalisations for each recording applied)

```
for (f in ST0[,unique(Filename)]){
  #show progress
  print(paste("Processing recording ",f,"... ",length(ST0[,unique(Filename)])-which(S
T0[,unique(Filename)]==f)," more recordings to process.",sep=""))
  #subset data table
  ST_temp=ST0[Filename==f]
  #import wave file
  ST_wave_temp=readWave(paste("Sound transmission/",f,sep=""))
  #compute spectrogram
  ST.spectro=spectro(ST_wave_temp,wl=1024,plot = F)
  #measure amplitudes in loop (find more efficient way)
  #for unknown reason amplitudes at 2m in no-horn recordings are unreasonably high (p
artly higher than at 4 m) - excluding them
  for (tp in ST_temp[
    !(Distance_m==2 & Method=="AR") | is.na(Distance_m)
    ,Time_signal]){
    if (ST_temp[Time_signal==tp,Type]=="ambient") {duration_temp_s=duration_ambient_s
    } else  if (ST_temp[Time_signal==tp,Type]=="signal") {duration_temp_s=duration_si
gnal_s}
    #measure amplitude at a bandwidth of 3 frequency bins (determined peak width from
Audacity)
    amplitude_temp=mean(ST.spectro$amp[c((frequency_bin-1):(frequency_bin+1))
                                       ,which.min(abs(ST.spectro$time-tp)):which.min
(abs(ST.spectro$time-(tp+duration_temp_s)))])
    #save data
    ST0[Filename==f & Time_signal==tp,Amplitude_dB:=amplitude_temp]
  }
}
```

```
## [1] "Processing recording ST_BP1_PC_US6_20200417_144258_with_AS.wav... 5 more reco
rdings to process."
## [1] "Processing recording ST_BP1_AR_US1_20190528_155435_with_AS.wav... 4 more reco
rdings to process."
## [1] "Processing recording ST_BP2_PC_US6_20200417_145915_with_AS.wav... 3 more reco
rdings to process."
## [1] "Processing recording ST_BP2_AR_US1_20190530_171346_with_AS.wav... 2 more reco
rdings to process."
## [1] "Processing recording ST_BP3_PC_US6_20200417_142524_with_AS.wav... 1 more reco
rdings to process."
## [1] "Processing recording ST_BP3_AR_US1_20190528_153419_with_AS.wav... 0 more reco
rdings to process."
```

Computing extinction distances and detection spaces

using log distance

```
ST0[,Distance_m_log:=log(Distance_m)]
```

calculate linear models for sound transmission

```
extinction0=ST0[!is.na(Amplitude_dB) & Type=="signal"
                ,.(intercept=coef(lm(Amplitude_dB~Distance_m_log))[1]
                   ,slope=coef(lm(Amplitude_dB~Distance_m_log))[2])
                ,.(Plot,Method,Direction)]
```

add ambient sound level

```
extinction1=merge(extinction0
                  ,ST0[!is.na(Amplitude_dB) & Type=="ambient",.(ambient=Amplitude_d
B),.(Plot,Method)]
                  ,by=c("Plot","Method"))
```

average out night-to-night differences that are irrelevant here

```
extinction1[,ambient_mean:=mean(ambient),Method]
```

calculate extinction distances/ranges

```
extinction1[,extinction_m:=exp((ambient_mean-intercept)/slope)]
```

summarize measures over all sites

```
extinction.mean=extinction1[,.(mean_range_m=mean(extinction_m)
                               ,min_max_range_m=paste(round(min(extinction_m)),"-",ro
und(max(extinction_m)))
                               ,ambient_mean_dB=mean(ambient))
                            ,.(Direction,Method)]
```

get maximum of mean ranges

```
extinction.mean[,max_mean_range_m:=max(mean_range_m)]
```

scale to 50 m for graphing

```
extinction.mean[,mean_range_scaled_50m:=round((mean_range_m/max_mean_range_m)*50,1)]
```

assign type of range

```
extinction.mean[,Type_range:="Ultrasound"]
```

check results with graphs

```
ggplot(ST0[Type=="signal"],aes(Distance_m_log,Amplitude_dB,color=Type))+
  geom_point()+
  stat_smooth(method="lm",se=F)+
  stat_smooth(method="lm",fullrange=T,lwd=0.3)+
  geom_hline(data=extinction1[Plot=="BP1",.(ambient_mean,Type="ambient",Method,Direct
ion,Plot)],aes(yintercept=ambient_mean,color=Type),lwd=1)+
  geom_hline(data=extinction1[Plot=="BP2",.(ambient_mean,Type="ambient",Method,Direct
ion,Plot)],aes(yintercept=ambient_mean,color=Type),lwd=1)+
  geom_hline(data=extinction1[Plot=="BP3",.(ambient_mean,Type="ambient",Method,Direct
ion,Plot)],aes(yintercept=ambient_mean,color=Type),lwd=1)+
  geom_vline(data=extinction1,aes(xintercept=log(extinction_m),alpha=extinction_m),lt
y=2)+
  scale_alpha_continuous(range=c(0.3,1))+
  facet_wrap(~paste(Plot,Method,Direction),ncol = 4)+
  scale_x_continuous(breaks = c(log(c(2,4,8,16,32,64,128,512))),labels =c(2,4,8,16,32
,64,128,512))+
  labs(x="Distance (m)")+
  theme_cowplot()+
  background_grid(major="xy")
```

```
## `geom_smooth()` using formula 'y ~ x'
```

```
## Warning: Removed 28 rows containing non-finite values (stat_smooth).
```

```
## `geom_smooth()` using formula 'y ~ x'
```

```
## Warning: Removed 28 rows containing non-finite values (stat_smooth).
```

```
## Warning: Removed 28 rows containing missing values (geom_point).
```

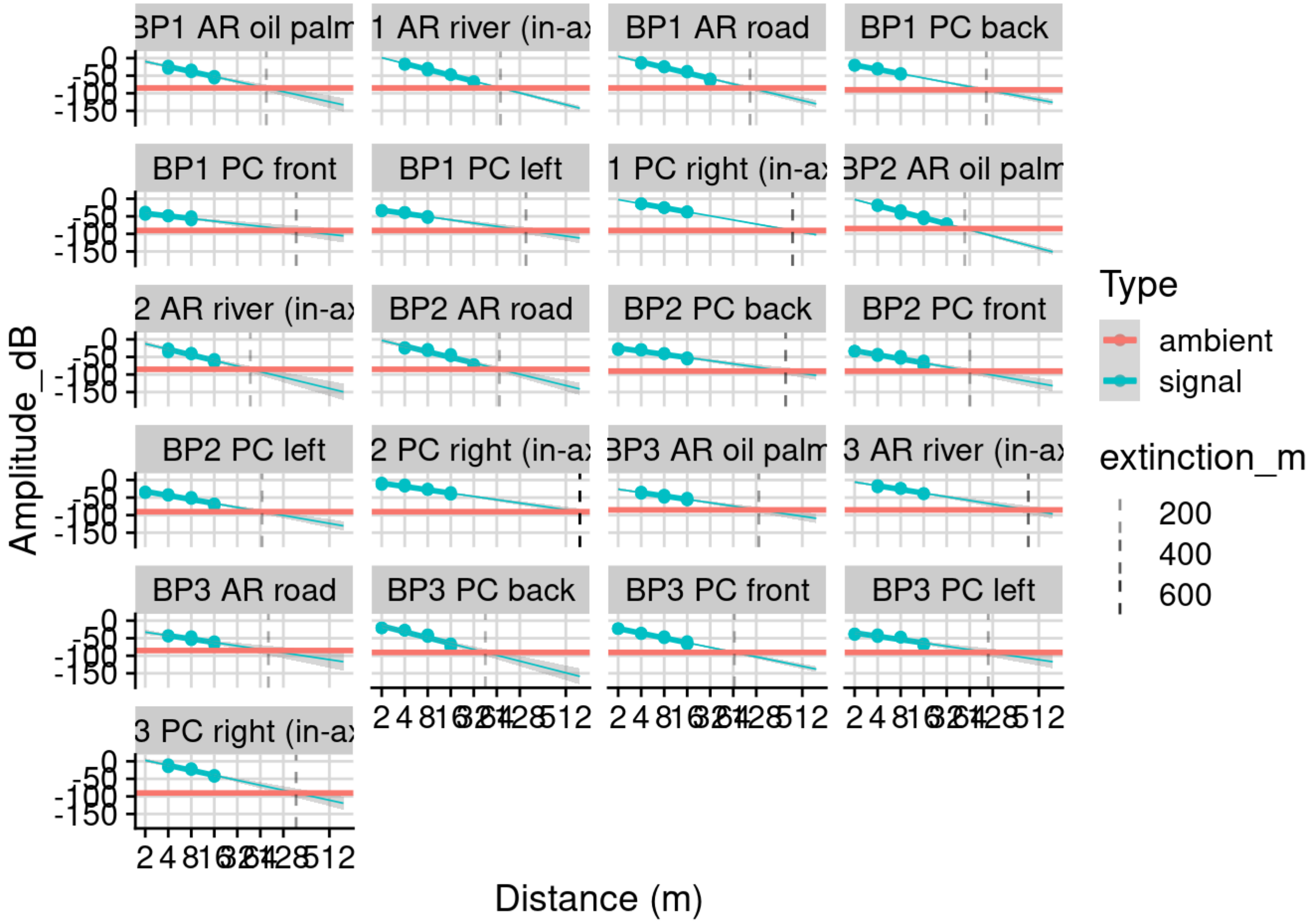


```
ggsave("Fig S1.png",width=10,height=10)
```

```
## `geom_smooth()` using formula 'y ~ x'
```

```
## Warning: Removed 28 rows containing non-finite values (stat_smooth).
```

```
## `geom_smooth()` using formula 'y ~ x'
```

```
## Warning: Removed 28 rows containing non-finite values (stat_smooth).

## Warning: Removed 28 rows containing missing values (geom_point).
```

```
ggsave("Fig S1.svg",width=10,height=10)
```

```
## `geom_smooth()` using formula 'y ~ x'
```

```
## Warning: Removed 28 rows containing non-finite values (stat_smooth).
```

```
## `geom_smooth()` using formula 'y ~ x'
```

```
## Warning: Removed 28 rows containing non-finite values (stat_smooth).

## Warning: Removed 28 rows containing missing values (geom_point).
```

Thermal ranges

```
thermal.range1=melt(plots0[,-c("X_WGS84","Y_WGS84")],id="Plot")
thermal.range1[grepl("oil_palm",variable),Direction:="oil palm"]
thermal.range1[grepl("river",variable),Direction:="river"]
thermal.range1[grepl("road",variable),Direction:="road"]
```

summarise thermal ranges

```
thermal.range2=thermal.range1[,.(mean_range_m=mean(value)
                                 ,min_max_range_m=paste(round(min(value)),"-",round(m
ax(value)))
                                 ,Type_range="Thermal"
                                 ,Method="PC")
                              ,.(Direction)]
```

bind all ranges in one table to manually generate Fig 4

```
ranges=rbind(extinction.mean,thermal.range2,fill=T)
ranges
```

```
##           Direction Method mean_range_m min_max_range_m ambient_mean_dB
##  1: river (in-axis)     AR    164.29768        47 - 374       -85.02035
##  2:        oil palm     AR     90.00376        55 - 138       -85.02035
##  3:            road     AR     85.66838        69 - 106       -85.02035
##  4: right (in-axis)     PC    450.51075       188 - 781       -90.26106
##  5:           front     PC    106.42650        64 - 189       -90.26106
##  6:            left     PC    111.14737        68 - 154       -90.26106
##  7:            back     PC    153.63150        45 - 310       -90.26106
##  8:        oil palm     PC     43.66667         39 - 46              NA
##  9:            road     PC     44.66667         19 - 66              NA
## 10:           river     PC     55.33333         38 - 84              NA
##     max_mean_range_m mean_range_scaled_50m Type_range
##  1:         450.5108                  18.2 Ultrasound
##  2:         450.5108                  10.0 Ultrasound
##  3:         450.5108                   9.5 Ultrasound
##  4:         450.5108                  50.0 Ultrasound
##  5:         450.5108                  11.8 Ultrasound
##  6:         450.5108                  12.3 Ultrasound
##  7:         450.5108                  17.1 Ultrasound
##  8:               NA                    NA    Thermal
##  9:               NA                    NA    Thermal
## 10:               NA                    NA    Thermal
```
