# Supplementary material for: Bat point counts: A novel sampling method shines light on flying bat communities
Source: Ecol Evol. 2021 Nov 30;11(23):17179–90. doi: 10.1002/ece3.8356 (PMC8668732; doi:10.1002/ece3.8356)
Supplement: Supplementary file 3 — Data S2 [file ECE3-11-17179-s004.docx]

## Text S1: In-depth analysis of rarefaction and extrapolation (RE) sampling curves

For generating abundance-based rarefaction and extrapolation sampling curves, we had to generate abundance data although they are are not directly derivable from the acoustic detections. For the main text, we chose a conservative number of sampled individuals for acoustic detections from bat point counts and ultrasound recordings based on the sum of the maximum number of simultaneous detections across species, as used in [Darras et al. (2018)](https://www.zotero.org/google-docs/?NrPKeB).

Here, we provide additional RE sampling curves based on the assumption that every detection is a different individual as a maximum, unrealistic estimate (Fig S2). This estimate is exacerbated by the long recording durations of automated recordings where identical bats would inevitably have been detected multiple times. Figure S2 shows that the efficiency of automated ultrasound recordings - as judged from the abundance-based species sampling curves - is uncertain and dependent on the abundance counting method; it explains why we did not discuss it further in the main text. The upper bound for the bat point count RE sampling curves also confirms that their estimates are more stable and similar to those from mist-nets, as in our main text results.

We caution that due to species-specific sound detection spaces [(Darras et al., 2016; Yip et al., 2017)](https://www.zotero.org/google-docs/?oOdM6j), intrinsic differences between the moving sampling area of the thermal scope, and the unknown sampling area of the vertical mist nets that occupy specific parts of the below-canopy space, we cannot compare abundances directly at a common sampling area. It is possible that the lower performance of mist nets with time might be due to the smaller mist net sampling area. The sampled species number could scale with increasing sampling areas, as for acoustic detection spaces in oil palm [(Darras et al., 2020)](https://www.zotero.org/google-docs/?ZbGzdN). However, in contrast to that study, our sampling durations were two to eight times longer, and the sampling areas of our methods were within the same order of magnitude. It seems more likely that given the high mobility of bats, our analysis of incidence-based RE sampling curves suffers less from potential biases arising from differences in sampling areas between methods, as the entire species pool would eventually enter the sampling area. Also, due to the high bat prevalence in our sites, the stress and potential abortions caused by prolonged capture times, and the fact that insectivorous bats considerably damage our nets by biting over time, we estimated that only four 12 m nets could be handled by a team of two persons. In effect, we standardised the sampling effort by using the same personnel as for carrying out bat point counts - and automated sound recorders are not comparable *per se*. Theoretically, acoustic detection spaces [(Darras et al., 2016)](https://www.zotero.org/google-docs/?dfv1o5) could also have been enlargened by using horns and additional microphones on the passive acoustic recorder, but we chose to equalise the number of microphones instead.

We provided additional RE sampling curves showing how sampling coverage is affected by increasing sampling effort (survey duration or number of individuals) and how sampling coverage determines species diversity (Fig S2). However, they have limited usefulness as for instance, maximal sampling coverage is rapidly reached for ultrasound recordings because most of the relatively few species that the method can detect are commonly detected. Finally, we show that including non-thermal point count detections to use a larger dataset does not substantially change the sampling effectiveness of point counts relative to the other methods (Fig S2).

##

# References

[Darras, K., Furnas, B., Fitriawan, I., Mulyani, Y., & Tscharntke, T. (2018). Estimating bird detection distances in sound recordings for standardizing detection ranges and distance sampling. *Methods in Ecology and Evolution*, *9*(9), 1928–1938. https://doi.org/10.1111/2041-210X.13031](https://www.zotero.org/google-docs/?Qw4Irx)

[Darras, K., Pütz, P., Fahrurrozi, Rembold, K., & Tscharntke, T. (2016). Measuring sound detection spaces for acoustic animal sampling and monitoring. *Biological Conservation*, *201*, 29–37. https://doi.org/10.1016/j.biocon.2016.06.021](https://www.zotero.org/google-docs/?Qw4Irx)

[Darras, K. F. A., Deppe, F., Fabian, Y., Kartono, A. P., Angulo, A., Kolbrek, B., Mulyani, Y. A., & Prawiradilaga, D. M. (2020). High microphone signal-to-noise ratio enhances acoustic sampling of wildlife. *PeerJ*, *8*, e9955. https://doi.org/10.7717/peerj.9955](https://www.zotero.org/google-docs/?Qw4Irx)

[Darras, Kevin Felix Arno. (2020). Bat point counts. *Open Science Framework*. https://doi.org/10.17605/OSF.IO/RQYH8](https://www.zotero.org/google-docs/?Qw4Irx)

[Yip, D., Leston, L., Bayne, E., Sólymos, P., & Grover, A. (2017). Experimentally derived detection distances from audio recordings and human observers enable integrated analysis of point count data. *Avian Conservation and Ecology*, *12*(1).](https://www.zotero.org/google-docs/?Qw4Irx)
