# Supplementary material for: Bat point counts: A novel sampling method shines light on flying bat communities
Source: Ecol Evol. 2021 Nov 30;11(23):17179–90. doi: 10.1002/ece3.8356 (PMC8668732; doi:10.1002/ece3.8356)
Supplement: Supplementary file 4 — Data S3 [file ECE3-11-17179-s009.docx]

## Data S3: Examples of near-infrared bat pictures

### *Cynopterus* sp. with visible hole in wing
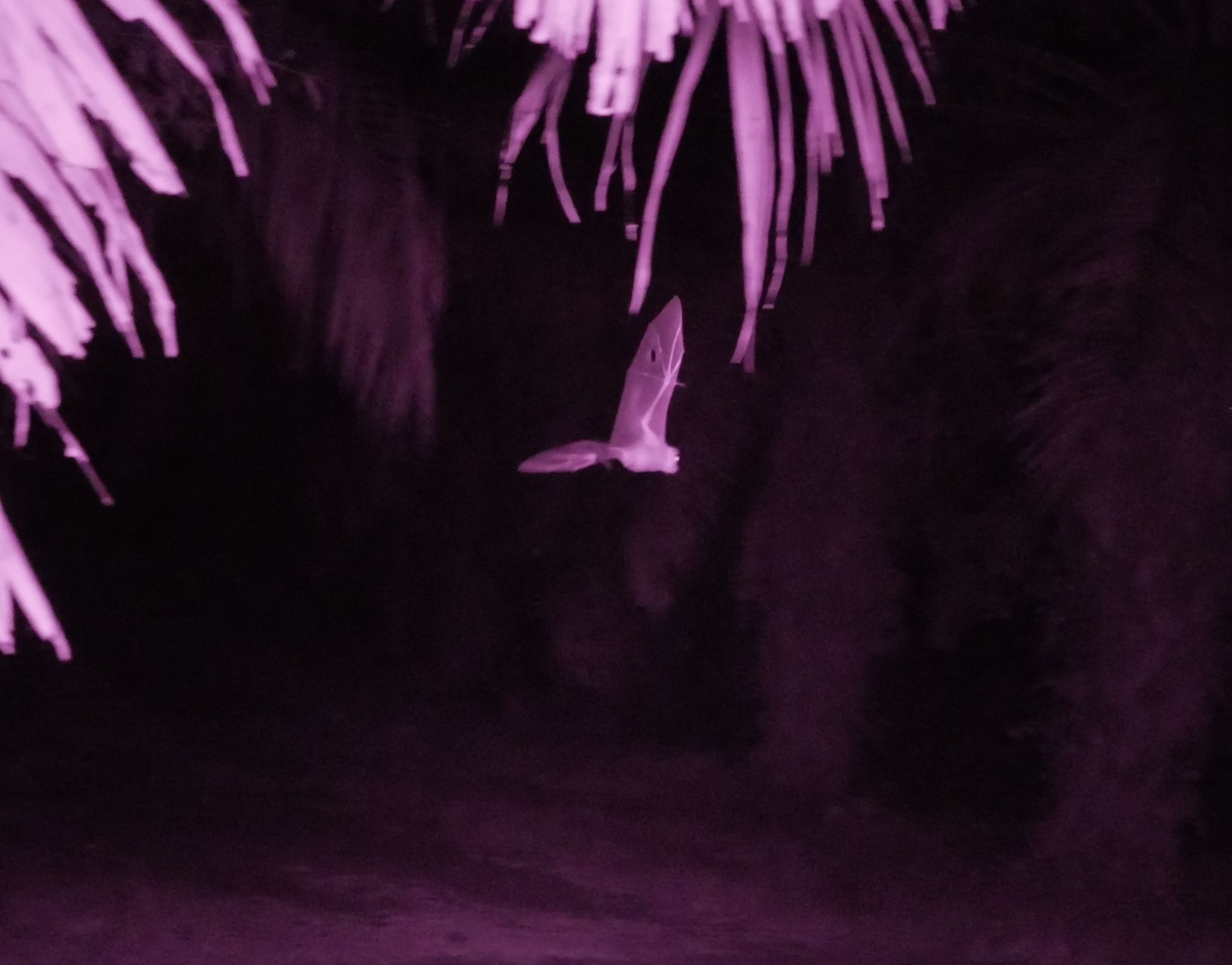


### ***Cynopterus* sp. with visible penis**
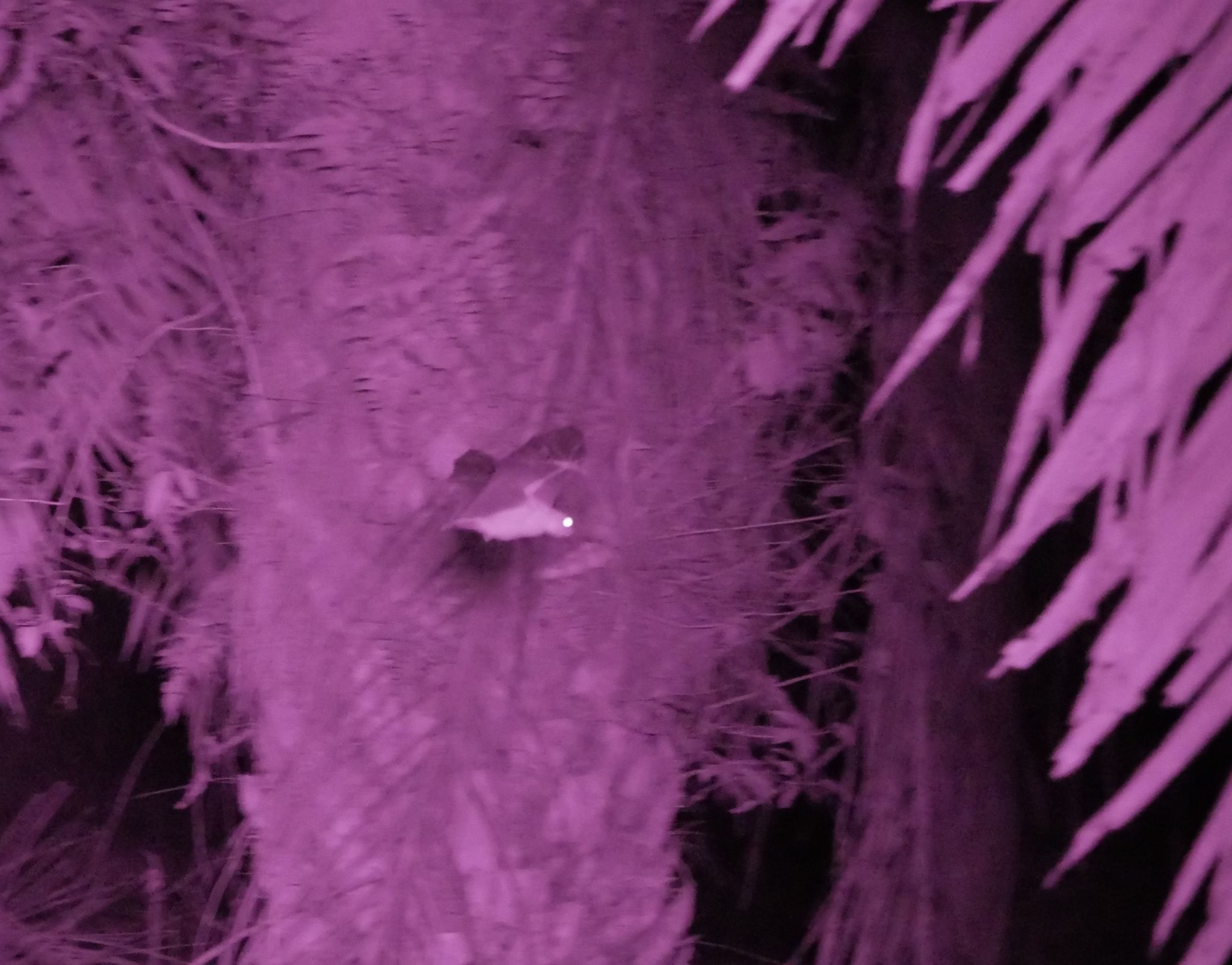


animated GIF:
<https://mfr.de-1.osf.io/render?url=https://osf.io/3utwg/?direct%26mode=render%26action=download%26mode=render>

### **Two echolocating bats encounter**
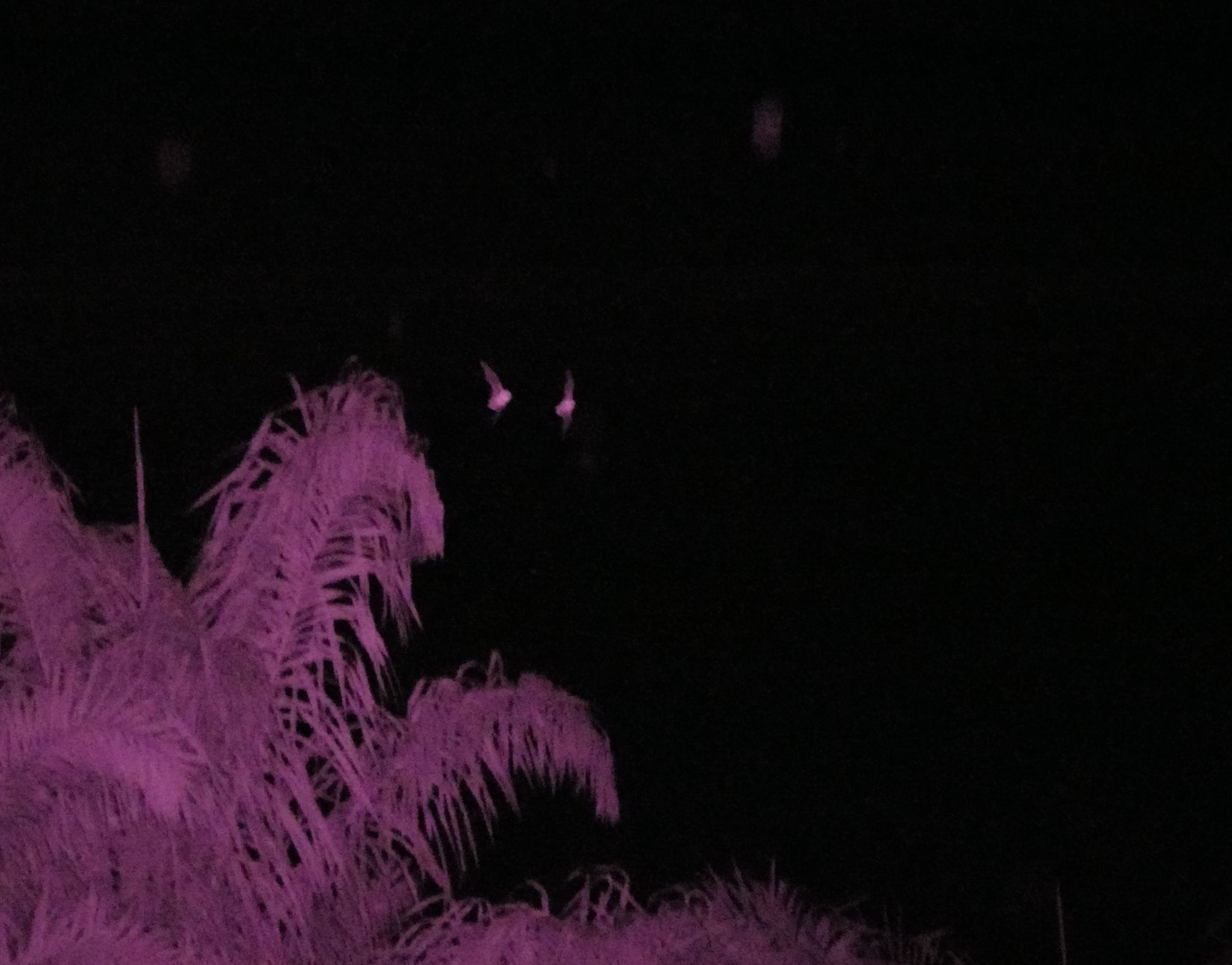


### Bat diving maneuver

animated GIF: <https://mfr.de-1.osf.io/render?url=https://osf.io/7phkf/?direct%26mode=render%26action=download%26mode=render>
Corresponding feeding buzz (note the concurrent shutter sounds and ca. 20 kHz call frequency drop after the feeding buzz):

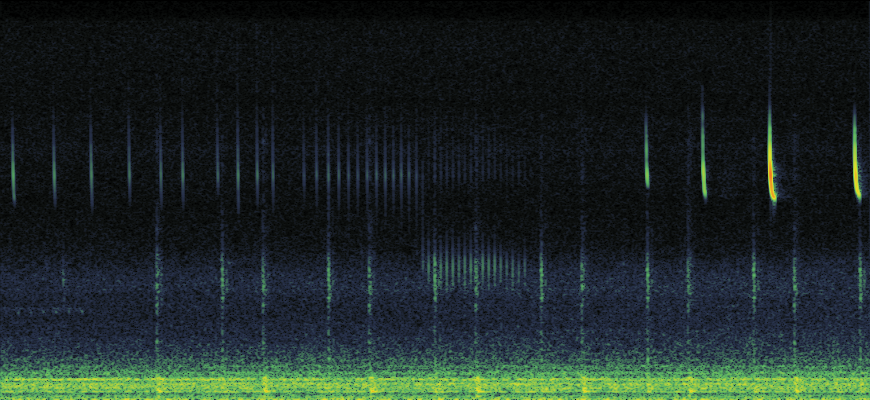

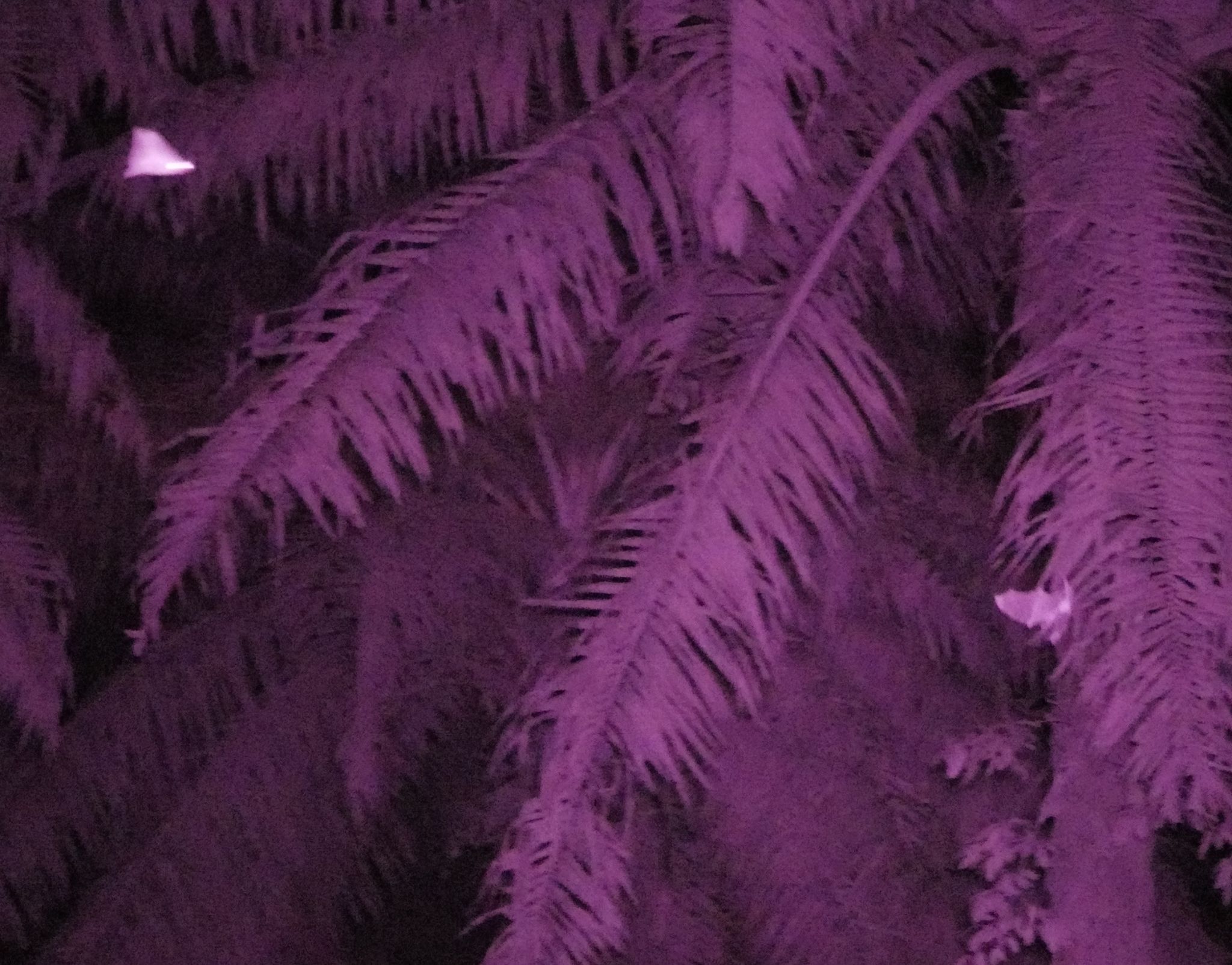


### Pteropodid pass with noticeable reflective eyes

animated GIF:
<https://mfr.de-1.osf.io/render?url=https://osf.io/dyf8w/?direct%26mode=render%26action=download%26mode=render>

### **Thermal scope video feed of a bat pass**

<https://mfr.de-1.osf.io/render?url=https://osf.io/sjxmh/?direct%26mode=render%26action=download%26mode=render>

### *Rhinolophus orbiculus*

Animated GIF:
<https://mfr.de-1.osf.io/render?url=https://osf.io/7yaqs/?direct%26mode=render%26action=download%26mode=render>

### Data repository

All photographs as well as animated GIFs are available in the Open Science Framework data repository:
<https://osf.io/rqyh8/>
